# Structures of the Omicron Spike trimer with ACE2 and an anti-Omicron antibody

**DOI:** 10.1126/science.abn8863

**Published:** 2022-02-08

**Authors:** Wanchao Yin, Youwei Xu, Peiyu Xu, Xiaodan Cao, Canrong Wu, Chunyin Gu, Xinheng He, Xiaoxi Wang, Sijie Huang, Qingning Yuan, Kai Wu, Wen Hu, Zifu Huang, Jia Liu, Zongda Wang, Fangfang Jia, Kaiwen Xia, Peipei Liu, Xueping Wang, Bin Song, Jie Zheng, Hualiang Jiang, Xi Cheng, Yi Jiang, Su-Jun Deng, H. Eric Xu

**Affiliations:** ^1^ The CAS Key Laboratory of Receptor Research, Shanghai Institute of Materia Medica, Chinese Academy of Sciences, Shanghai 201203, China.; ^2^ Shanghai Jemincare Pharmaceuticals Co., Ltd., Shanghai 201203, China.; ^3^ University of Chinese Academy of Sciences, Beijing 100049, China.; ^4^ The Shanghai Advanced Electron Microscope Center, Shanghai Institute of Materia Medica, Chinese Academy of Sciences, Shanghai 201203, China.; ^5^ State Key Laboratory of Drug Research, Shanghai Institute of Materia Medica, Chinese Academy of Sciences, Shanghai 201203, China.; ^6^ Immunological Disease Research Center, Shanghai Institute of Materia Medica, Chinese Academy of Sciences, .Shanghai 201203, China.; ^7^ School of Life Science and Technology, ShanghaiTech University, 201210 Shanghai, China.

## Abstract

The SARS-CoV-2 Omicron variant has become the dominant infective strain. We report the structures of the Omicron spike trimer on its own or in complex with ACE2 or an anti-Omicron antibody. Most Omicron mutations are located on the surface of the spike protein, which change binding epitopes to many current antibodies. In the ACE2 binding site, compensating mutations strengthen RBD binding to ACE2. Both the RBD and the apo form of the Omicron spike trimer are thermodynamically unstable. An unusual RBD-RBD interaction in the ACE2-spike complex supports the open conformation and further reinforces ACE2 binding to the spike trimer. A broad-spectrum therapeutic antibody, JMB2002, which has completed a Phase 1 clinical trial, maintains neutralizing activity against Omicron. JMB2002 binds to RBD differently from other characterized antibodies and inhibits ACE2 binding.

The Omicron variant of SARS-CoV-2, the causative virus of COVID-19, was initially reported from South Africa in November 2021, and quickly became the dominant strain worldwide (*1*). Phylogenetic tree analyses reveal that Omicron evolved independently from previous variants of concerns (VOC), including the predominant Alpha, Beta, Gamma, and Delta variants (Fig. 1A) (*2*–*5*). Compared to the original wildtype (WT) strain of SARS-CoV-2, Omicron has 60 amino acid mutations, of which 37 mutations are in the spike protein, the target of most COVID-19 vaccines and therapeutic antibodies (Fig. 1B). This high variation is reflected in different behavior with the Omicron variant showing enhanced transmission, antibody evasion, and vaccine resistance (*6*–*8*).

**Fig. 1.**
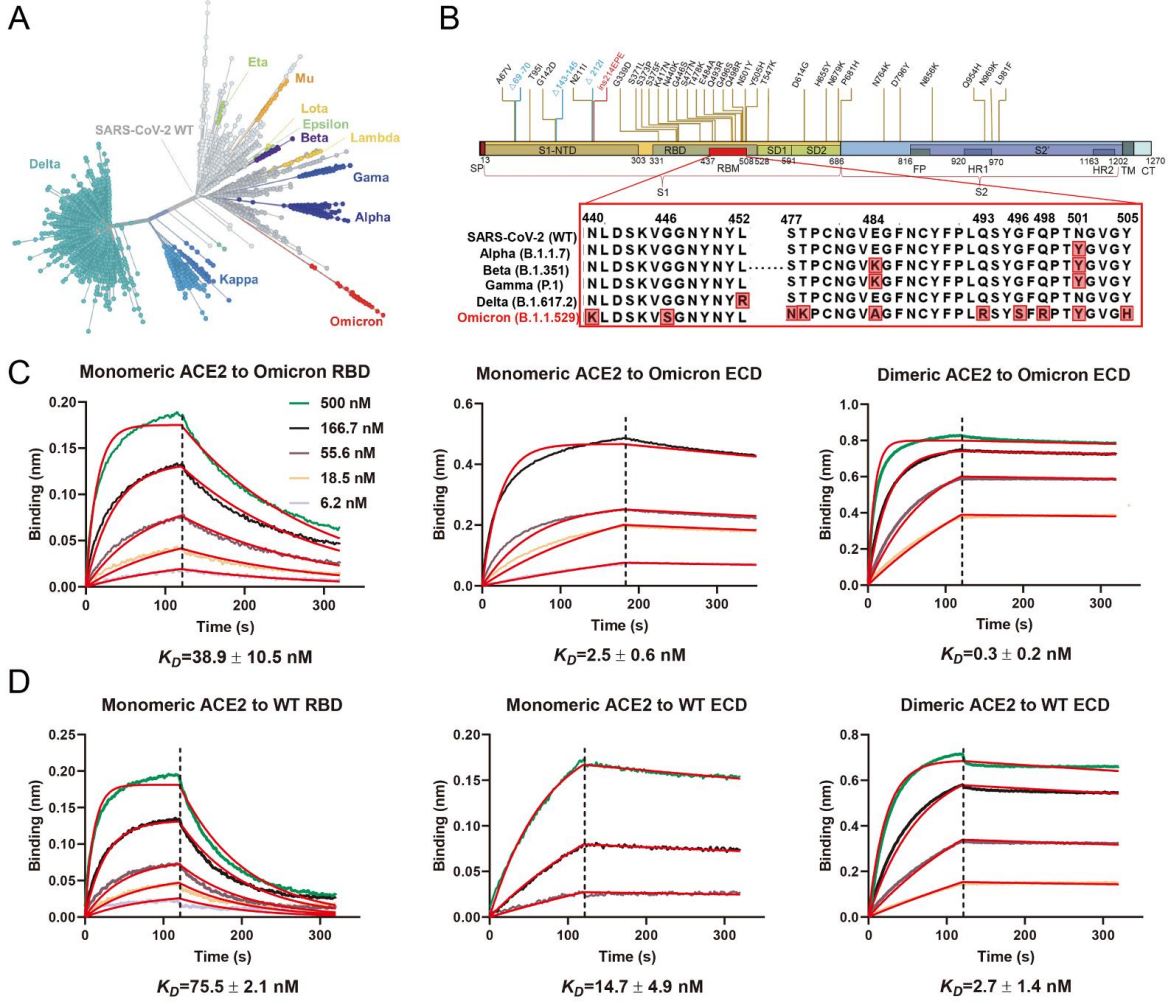
High-affinity binding of the SARS-CoV-2 Omicron spike protein with human ACE2. (**A**) Phylogeny of the SARS-CoV-2 variants. Variants of concern and variants of interest are labeled on the graph, the number of spike protein mutations are positively correlated with the distance from the original strain. (**B**) Schematic of Omicron spike protein domain architecture. The mutations of Omicron spike protein are labeled with different colors (blue for deleting mutation, red for inserting mutation). Mutations in RBM are compared with wildtype SARS-CoV-2 and four other variants of concern strains. SP, signal peptide; RBD, receptor binding domain; RBM, receptor binding motif; SD1, subdomain 1; SD2, subdomain 2; FP, fusion peptide; HR1, heptad repeat 1; HR2, heptad repeat 2; TM, transmembrane region; CT, cytoplasmic tail. (**C** and **D**) Binding of Omicron and WT spike trimer and RBD to ACE2 determined by BLI.

To study the mechanism for Omicron’s enhanced transmission, we first biochemically characterized the interactions of the SARS-CoV-2 receptor ACE2 with the trimer of the spike extracellular domain (ECD) from Omicron and the original WT strain, both of which contain proline substitutions (2P or 6P) and a mutated furin cleavage site to stabilize the prefusion conformation (*9*, *10*). Monomeric human ACE2 bound to immobilized Omicron trimeric spike protein with approximately 6-fold higher affinity (K_D_=2.5 ± 0.6 nM) than WT spike trimer (K_D_=14.7 ± 4.9 nM). The dimeric human ACE2 bound to immobilized biotinylated Omicron spike trimer (K_D_=0.3 ± 0.2 nM) with approximately 9-fold higher avidity than WT (K_D_=2.7 ± 1.4 nM) (Fig. 1, C and D). We then studied the interactions of ACE2 with monomeric receptor binding domain (RBD) from Omicron and WT strains. Monomeric human ACE2 bound to immobilized Omicron RBD (K_D_=38.9 ± 10.5 nM) with approximately 2-fold higher affinity than WT RBD (K_D_=75.5 ± 2.1 nM) (Fig. 1, C and D). The enhanced interaction of Omicron spike and RBD proteins with human ACE2 is consistent with previously published data (*11*), and may contribute to the increased infectivity of the Omicron variant.

To determine the structural basis of higher affinity of the Omicron spike trimer for ACE2, we solved the structure of the ACE2-Omicron spike trimer complex at a global resolution of 2.77 Å (table S1). Despite an excess of ACE2 (molar ratio of 3.2 ACE2 to 1 spike trimer; fig. S1A), we only observed strong density for one ACE2 bound to one RBD from the spike trimer in the open “up” conformation (Fig. 2A and fig. S2). The other two RBDs, with clear density, are in the closed “down” conformation. Particle classification revealed that the majority of picked particles (~70%) do not have ACE2 bound. We also determined the structure of this apo Omicron spike trimer at a global resolution of 2.56 Å (fig. S2 and table S1). All three RBDs are in the closed down conformation but they are less visible in the high-resolution map (2.56 Å; fig. S3A), yet become more visible in lower resolution maps (4.5 Å and 6.5 Å; fig. S3, B and C). This contrasts with the clear visibility of the three RBDs in the ACE2-Omicron spike complex in a high-resolution map (2.56 Å; Fig. 2A), indicating that the RBD in the apo form is more dynamic and ACE2 binding likely stabilizes the conformation of the three RBDs. Thermal shift assays at pH7.4 revealed that the Omicron and WT RBD have single melting temperatures of 45.7°C and 51.0°C respectively (fig. S1C), indicating that the Omicron RBD is less stable than the WT RBD. In contrast, both the Omicron and WT spike trimer displayed two melting temperatures (fig. S1D), with the high Tm corresponding to the dissociation of the spike trimer and the low Tm corresponding to unfolding of the RBD. The melting temperature profile of WT spike trimer is similar to previous reports (*10*, *12*). Tm1 of both Omicron and WT spike trimer is similar to the respective Tm for the isolated RBD (fig. S1, C and D), indicating that the Omicron RBD within the context of the spike trimer remains less stable than the WT RBD. We further confirmed the highly flexible nature of the Omicron RBD by performing hydrogen-deuterium exchange mass spectrometry (HDX), which showed that the Omicron spike trimer has an overall higher rate of HDX (fig. S4), particularly in the RBD region, consistent with its lower thermal stability.

**Fig. 2.**
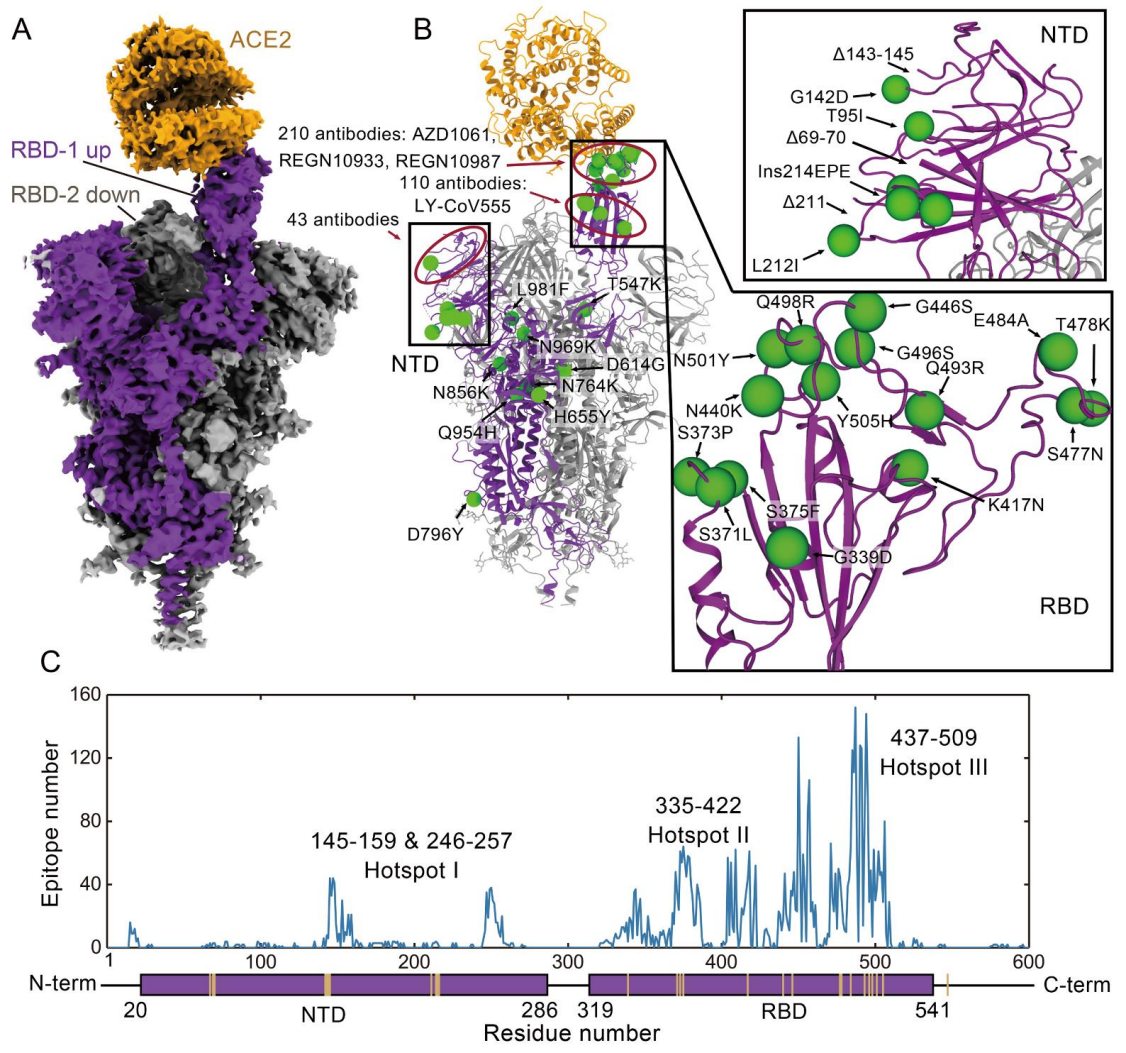
The structure of ACE2 bound Omicron spike trimer complex and epitopes of current antibodies. (**A**) Cryo-EM density of the ACE2-Omicron spike trimer complex. (**B**) The overall structure of the ACE2-Omicron spike trimer complex and the locations of Omicron mutations. Epitope hotspots are highlighted in red circles with the number of antibodies indicated next to the epitopes. (**C**) The histogram of epitope corresponding with residue numbers. Each epitope was counted if more than 3 heavy atoms of this residue are closer than 5 Å with the antibody. The PDB IDs and corresponding epitopes were summarized in table S2 in the supplementary materials.

Mapping the 37 mutations onto the up protomer of the ACE2 bound spike trimer reveals that most mutations are located on the surface of the spike protein, with many of them in known epitopes of therapeutic antibodies (Fig. 2B). We grouped the surface mutations into 3 hotspots (Fig. 2C and table S2). Eight mutations in the NTD (hotspot I) would affect the structures of the epitopes for a number of antibodies, for example, Δ143-145 would remove the epitope for the 4A8 antibody (*13*). 15 mutations are in the RBD, which contains the ACE2 binding site as well as the epitopes for 90% of antibodies induced by infection or vaccination. 10 of these mutations are in the RBM (hotspot II) and 5 are near the core structure domain (hotspot III) (Fig. 2C). Hot spot II encompasses the epitopes for therapeutic antibodies, AZD1061, REGN10987 and REGN10933 and hotspot III overlaps the epitope for LY-CoV555 (Fig. 2B) (*14*–*16*).

Local refinement of the ACE2-RBD region produced a high-quality map at 2.57 Å resolution, which allowed unambiguous building of the ACE2-RBD complex (Fig. 3A, table S1, and fig. S2). Though their RBDs differ at 15 residues, the overall structure of the Omicron ACE2-RBD complex is similar to two high resolution X-ray structures of the WT ACE2-RBD complex (PDB codes: 6LZG and 6M0J (*17*, *18*)), with the Cα atoms of the whole RBD deviating by less than 0.4 Å (Fig. 3B and table S3). We do see local differences at the ACE2-RBD interface; the Omicron RBD forms extra interactions with ACE2, including interactions from RBD mutations S477N, Q493R, Q496S, Q498R, and N501Y to ACE2 (Fig. 3C). In particular, the side chain of S477N forms two extra hydrogen bonds with S19 of ACE2; the Q498R mutation forms two additional hydrogen bonds with Q42 and D38 from ACE2; and the N501Y mutation forms extensive packing interactions with ACE2 residues Y41 and K353. These additional interactions may compensate for the loss of polar interactions between WT RBD and ACE2 caused by RBD mutations K417N and E484A (Fig. 3, C and D), consistent with the enhanced affinity of the Omicron RBD with ACE2 (Fig. 1, C and D).

**Fig. 3.**
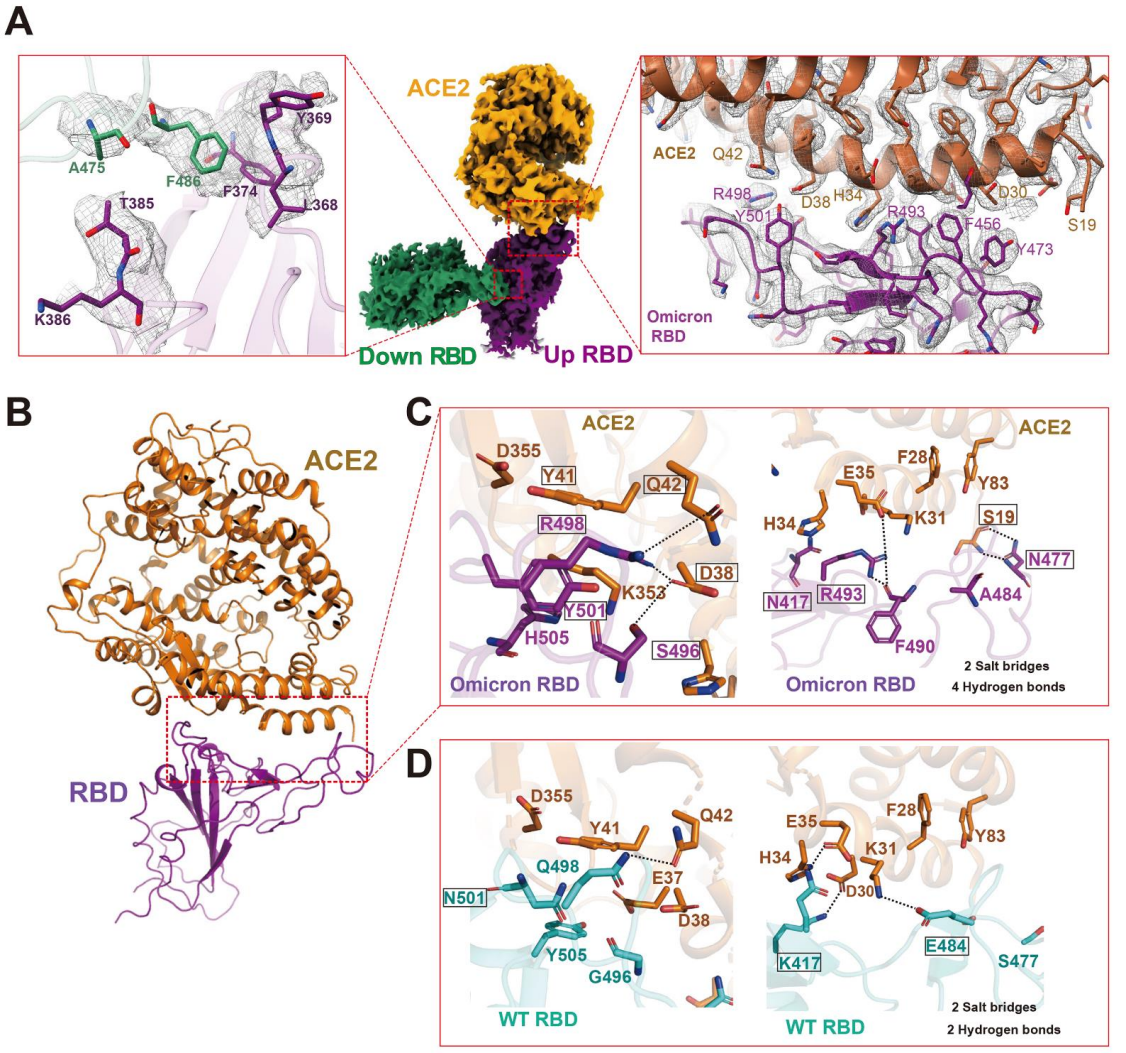
Structural analysis of Omicron RBD and ACE2. (**A**) Cryo-EM density of Omicron RBD-RBD-ACE2 interface. ACE2 is colored in orange. The ACE2 bound RBD, also named up RBD is in purple. The down RBD, which directly binds to up RBD is in green. Left panel, a close-up view of RBD-RBD interaction. Middle panel, overall cryo-EM density of down RBD-RBD-ACE2 region. Right panel, the ACE2-RBD binding interface. Residues are shown in sticks with the correspondent cryo-EM density represented in mesh. (**B**) An overall structural model of Omicron RBD-ACE2 bound region. (**C**) Zoomed-in view of Omicron RBD-ACE2 with hydrogen bonds interactions. (**D**) Detailed hydrogen bonds interactions in WT RBD-ACE2 interfaces with the same view as in panel B. WT RBD is sea blue, Omicron RBD is purple, and ACE2 is orange. Hydrogen bond or salt bridge interactions are in dotted lines.

We observe RBD-RBD interactions from one of the two down RBDs to the up RBD (Fig. 3A), with the interface comprised by residues A475 and F486 from the down RBD and residues L368, F374 and T385 from the up RBD (Fig. 3A and fig. S5A). Structure comparison reveals that the RBD-RBD interface is not observed within the WT spike trimer because of movement of a loop (residues 368-374), caused by Omicron RBD mutations S371L, S373P, G339D, and S375F, which are in hotspot III (Fig. 2C and fig. S5A). This RBD-RBD interaction may stabilize the up conformation of the RBD that promotes ACE2 binding. In addition, the Omicron mutations S371L, S373P and S375F are located at the entrance to the fatty acid binding pocket in the WT RBD (*19*), and these Omicron mutations distort the fatty acid binding pocket (fig. S5B), thus destabilizing the RBDs in the all closed-down conformation. Consistent with involvement of spike dynamics, ACE2 binds to the Omicron spike trimer with 6-9 fold higher avidity than to the WT spike trimer, but binds to the Omicron RBD monomer with 2- fold higher affinity than to WT RBD. (Fig. 1, C and D). We suggest that in addition to destabilization of RBDs in the closed conformation, the RBD-RBD interactions that stabilize one RBD in the open up conformation within the spike trimer, may act together with the compensating mutations in the ACE2 binding site to contribute to the higher affinity of Omicron and this likely plays a role in its higher infectivity.

We had previously discovered an antibody, JMB2002, which showed potent efficacy against the WT SARS-CoV-2 in cell-based models as well as in a rhesus monkey model (*20*). JMB2002 has completed Phase I clinical trial in healthy donors with excellent safety and pharmacokinetic properties and has been approved for a clinical trial in the USA (IND 154745). We evaluated the binding of JMB2002 to WT and Omicron spike trimers. JMB2002 Fab bound the Omicron spike trimer with ~4-fold increased affinity (K_D_=3.2 ± 3.0 nM) compared to WT spike trimer (K_D_=12.2 ± 11.6 nM), while JMB2002 IgG showed similar avidity for Omicron spike trimer (K_D_=0.4 ± 0.1 nM) and WT (K_D_=0.5 ± 0.3 nM) (Fig. 4, A to D). Furthermore, JMB2002 was able to directly inhibit the binding of ACE2 to the Omicron spike trimer with an IC_50_ of 1.8 nM (Fig. 4E). In pseudovirus neutralization assays, JMB2002 effectively blocked the entry of Omicron pseudovirus into human ACE2 expressing cells in addition to its blocking of the WT pseudovirus (Fig. 4F and fig. S6A). JMB2002 was also able to neutralize a number of VOCs, including variants of Alpha, Beta, and Gamma, but not Delta (fig. S6, B to E).

**Fig. 4.**
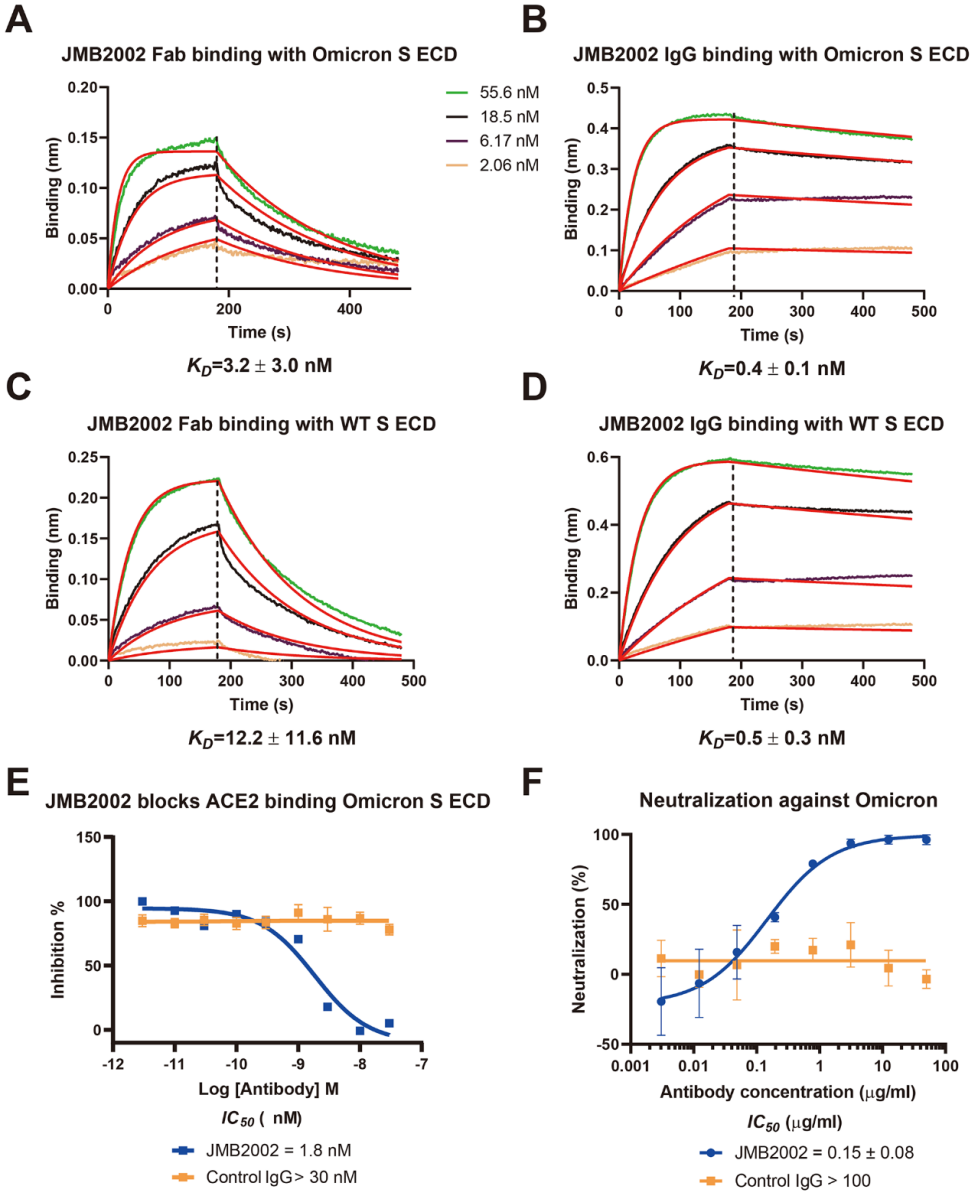
Inhibition of ACE2 binding to the spike trimer by an anti-Omicron antibody. (**A** and **C**) Binding of JMB2002 Fab to Omicron and WT spike trimer. (**B** and **D**) Binding of JMB2002 IgG to Omicron and WT spike trimer. (**E**) Direct inhibition of ACE2 binding to the Omicron spike trimer by JMB2002. (**F**) Inhibition of the pseudo virus of Omicron by JMB2002.

To reveal the basis of JMB2002 inhibition of Omicron, we solved the structure of the Omicron spike trimer bound to a Fab from JMB2002 at a global resolution of 2.69 Å (Fig. 5A, table S1, and figs. S7 and S8). To stabilize the constant regions of Fab, we used a nanobody that binds to the interface between the variable and constant regions of the light chain (*21*). The EM density map reveals the binding of 2 Fab molecules to two RBDs (one up and one down) of the trimeric spike (Fig. 5, A and B). The overall structure of the spike trimer in the Fab-bound complex is very similar to that of the ACE2-bound complex, with an RMSD of 1.0 Å over all Cα atoms of the spike trimer, including the unusual RBD-RBD dimer configuration (fig. S9, A and B).

**Fig. 5.**
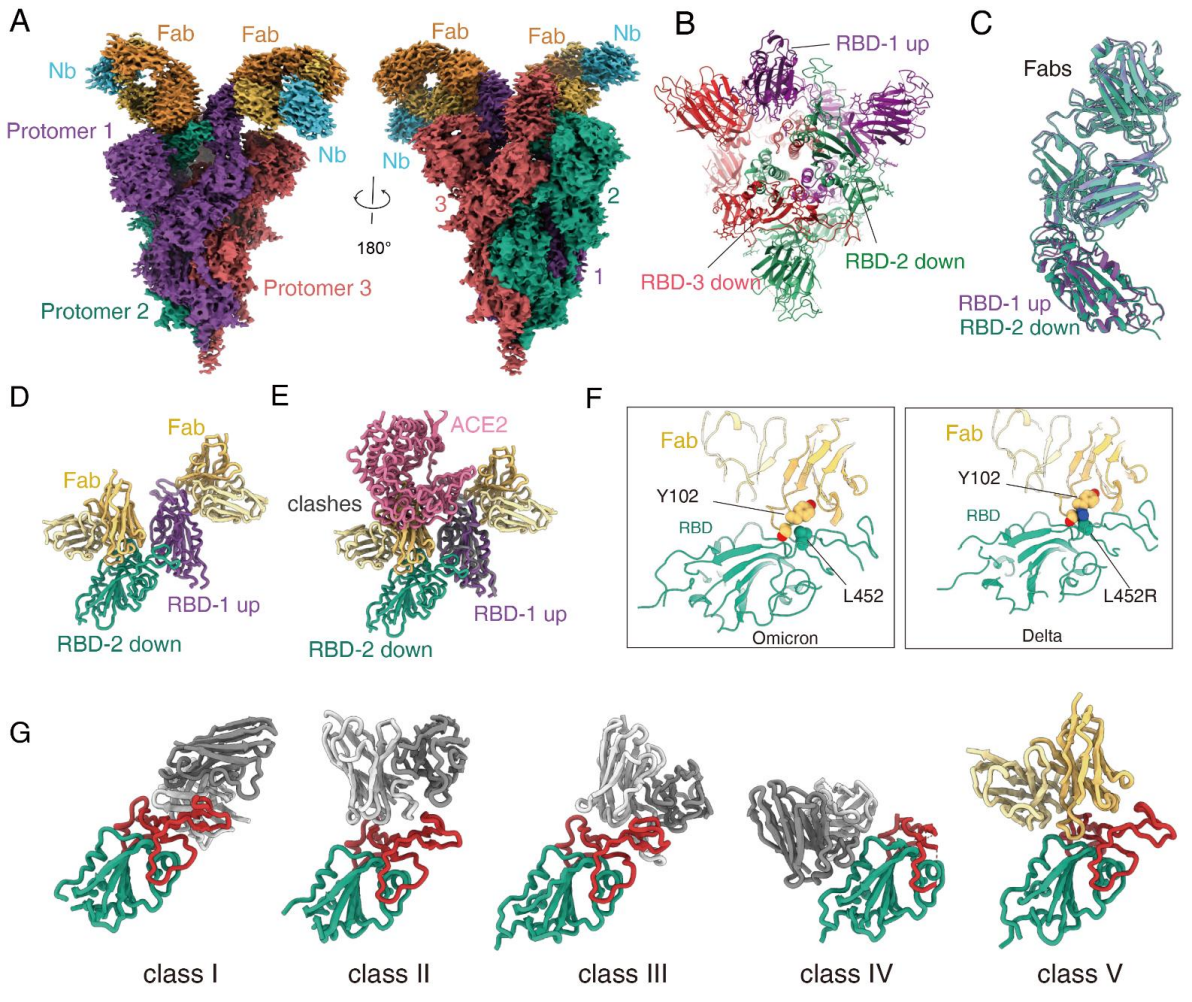
Structure of Omicron spike trimer with antibody JMB2002. (**A**) Cryo-EM density map of the Fab-bound Omicron spike trimer showed with two front views. (**B**) Top view of Fab-bound Omicron spike trimer complex model with fab and nanobody hidden. (**C**) Superposition of the Fab bound RBD-1 and RBD-2. **(D)** The structure of the Fab bound RBD-1 and RBD-2. (**E**) Superposition of the ACE2-bound and Fab-bound RBD-1 shows the Fab binding to RBD-2 inhibits ACE2 binding to RBD-1. (**F**) Left panel, L452 residue from Omicron RBD interacts with Fab. Right panel, the Delta variant L452R mutation clashes the Fab binding. (**G**) Binding modes of 4 representative 4 classes of antibody that neutralize SARS-CoV-2. PDB codes: class I, 7CM4; class II, 7CHF; class III, 7K90; class IV, 6WPS. The JMB2002Fab in the Omicron S protein structure shows distinct binding modes from other 4 classes of antibodies.

Within the Fab-spike trimer structure, both Fabs bind to the same region in their respective RBD (Fig. 5, C and D). Local refinement of the Fab bound RBD structure generated a density map to a resolution of 2.47 Å (figs. S7G and S8D), which provides detailed interactions between Fab and RBD. The Fab binding site does not overlap with the ACE2 binding site (fig. S9C). However, in the context of the trimer, Fab binding to the down RBD would clash with ACE2 binding to the up RBD (Fig. 5E), consistent with direct inhibition of ACE2 binding to the Omicron spike trimer by JMB2002 (Fig. 4E).

Particle classification also revealed two additional antibody-bound complexes at a global resolution of 2.92 Å and 3.18 Å, respectively (figs. S7 and S8). One of the two complexes has the spike trimer with one-up RBD bound to one Fab and two-down RBDs in the apo state (fig. S8A). The other complex contains the spike trimer with two-up RBDs and one down RBD, with each RBD bound to one Fab (fig. S8C). The up-down RBD-RBD interactions within the spike trimer are conserved in these complexes. The diverse configuration and stoichiometry ratio of the spike trimer bound to the antibody further highlight the conformation flexibility of the Omicron spike RBD. The ability of the spike trimer to bind to three Fab molecules provides additional basis for the potency of JMB2002 against Omicron.

The L452R mutation in the Delta variant is at the center of the binding epitope of JMB2002 and this mutation would clash with Y102 from the heavy chain of the Fab (Fig. 5F), thus providing an explanation for its loss of potency against the Delta variant. In addition, the binding site of the JMB2002 Fab on the RBD is distinct from the epitopes for previously defined class I to class IV antibodies (Fig. 5G) (*22*), thus JMB2002 represents a new class of antibody against the spike trimer.

In this paper, we report biochemical characterization of the Omicron spike trimer and its binding to ACE2. Our data reveal that the Omicron RBD is less stable and more dynamic than the WT RBD, and the Omicron spike trimer has 6-9 fold increased affinity for binding to ACE2. We further solved the structures of the Omicron spike trimer in the apo state, or bound to ACE2 or an anti-Omicron antibody. The ACE2 bound structure reveals that the Omicron spike trimer contains an unusual RBD-RBD interaction and extra interactions in the ACE2-RBD interface, both of which contribute to the higher affinity of ACE2 to the Omicron spike trimer. Structural analysis of the Omicron spike trimer also provides a mechanistic basis for the ability of Omicron to escape most therapeutic antibodies and reduce the efficacy of vaccinations.

In addition, our structures of antibody-bound Omicron spiker trimer uncover a distinct mode of antibody binding to the spike trimer, in which the unusual RBD-RBD configuration is preserved. The binding epitope of this broad-spectrum antibody is different from previous anti-SARS-CoV-2 antibodies, therefore opening a new venue for antibody drug discovery targeting various strains of SARS-CoV-2, including Omicron.

## Supplementary Materials

### This PDF file includes:

Materials and Methods Figs. S1 to S10 Tables S1 and S3 References (*23*–*36*) Table S2 MDAR Reproducibility Checklist

### Other Supplementary Material for this manuscript includes the following:

MDAR Reproducibility Checklist
