# Supplementary material for: Structures of the Omicron Spike trimer with ACE2 and an anti-Omicron antibody
Source: Science. 2022 Feb 8:eabn8863. doi: 10.1126/science.abn8863 (PMC8939775; doi:10.1126/science.abn8863)
Supplement: Supplementary file 1 — Materials and Methods Figs. S1 to S10 Tables S1 and S3 References (23–36) Table S2 MDAR Reproducibility Checklist [file science.abn8863_sm.pdf]

Science
AAAS

Supplementary Materials for

Structures of the Omicron Spike trimer with ACE2 and an anti-Omicron antibody

Wanchao Yin *et al.*

Corresponding authors: Wanchao Yin, wcyin@simm.ac.cn;
Su-Jun Deng, dengsujun@jemincare.com; H. Eric Xu, eric.xu@simm.ac.cn

DOI: 10.1126/science.abn8863

The PDF file includes:

Materials and Methods
Figs. S1 to S10
Tables S1 and S3
References

Other Supplementary Material for this manuscript includes the following:

Table S2 (Excel)
MDAR Reproducibility Checklist (PDF)

Materials and Methods

Phylogenetic tree generation

The phylogenetic tree of SARS-CoV-2 were carried out in the nextstrain Server in December 22th, 2021 (https://nextstrain.org/ncov/gisaid/global). The tree is shown as unrooted format and colored by clade.

Protein expression and purification

The N-terminal peptidase domain of human ACE2 (residues Ser19–Asp615) with an N-terminal GP67 signal peptide for secretion and a C-terminal 8×His tag for purification was inserted into a modified pFastBac vector (Invitrogen). All constructs were generated using the Phanta Max Super-Fidelity DNA Polymerase (Vazyme Biotech Co.,Ltd) and verified by DNA sequencing (Genewiz). Hi5 cell cultures were grown in ESF 921 serum-free medium (Expression Systems) to a density of 2-3 million cells per ml and then infected with baculoviruses for human ACE2 at a multiplicity of infection (m.o.i.) of about 5. The supernatant of cell culture containing the secreted peptidase domain of human ACE2 was collected 60 h after infection, and then bound to Ni-NTA beads (Smart-Lifesciences). After wash with buffer without imidazole, the protein was eluted with buffer containing 300 mM imidazole, concentrated and then size-separated by a Superdex 200 Increase10/300 GL column in 25 mM HEPES pH 7.4, 150 mM sodium chloride. The fractions for the peptidase domain of human ACE2 were collected, concentrated to about 2 mg/ml, and stored at − 80 °C until use.

The SARS-CoV-2 Spike ECD and RBD proteins were all purchased from Sino Biological Inc., including Omicron spike ECD trimer (Cat: 40589-V08H26: with furin cleavage site mutants and 2P mutants, that is R682G, R683S, R685S, K986P and V987P), Omicron spike RBD protein (Cat: 40592-V08H121), WT spike ECD trimer (Cat: 40589-V08H8: with D614G mutants, furin cleavage site mutants and 6P

mutants, that is D614G, R682G, R683S, R685S, F817P, A892P, A899P, A942P, K986P and V987P), WT spike RBD protein (Cat: 40592-V08H), Delta spike ECD trimer (Cat: 40589-V08H10: with furin cleavage site mutants and 6P mutants, that is R682G, R683S, R685S, F817P, A892P, A899P, A942P, K986P and V987P), Delta spike RBD protein (Cat: 40592-V08H90). According to the description from Sino Biological, all three ECD proteins were expressed with the bacteriophage T4 fibritin trimerization motif and a polyhistidine tag at the C-terminus, while all RBD proteins were expressed with a polyhistidine tag at the C-terminus.

Nanobody (Nb) (the amino acid sequence:
MGSQVQLQESGGGLVQPGGSLRLSCAASGRTISRYAMSWFRQAPGKEREFVAVA
RRSGDGAFYADSVQGRFTVSRDDAKNTVYLQMNSLKPEDTAVYYCAIDSDTFYSG
SYDYWGQGTQVTVSSHHHHHHEPEA) with a C-terminal His6 tag was expressed in the periplasm of *E. coli* strain BL21(DE3) bacteria (NEB). Cultures of 2 L cells were grown to OD600 = 0.8 at 37 °C, 180 r.p.m. in 2×YT media containing 100 μg/mL ampicillin. Then 0.1 mM IPTG was added to the medium to induce protein expression at 28 °C, 180 r.p.m. for another 8 h. Cells were harvested by centrifugation (5316 g, 30 min) and disturbed in ice-cold buffer (20 mM HEPES pH 7.4, 100 mM NaCl), then centrifuged to remove cell debris. Nb was purified by nickel affinity chromatography as previously described followed by size-exclusion chromatography using a HiLoad 16/600 Superdex 75 column. Selected fractions of Nb were finally concentrated to ~2 mg/ mL with 10% glycerol and kept frozen at -80 °C for further use. The quality of purified proteins was assessed by SDS-PAGE.

Protein complex formation

The amino acid sequences for the JMB2002Fab are listed as below:

Light chain:

DIQMTQSPSSLSASVGDRVTITCRASQGISSWLAWYQQKPGKAPKLLIYDASNLET
GVPSRFSGSGSGTDFTFTISSLQPEDIATYYCQQYDNLPLTFGGGTKVEIKRTVAAP

SVFIFPPSDEQLKSGTASVVCLLNNFYPREAKVQWKVDNALQSGNSQESVTEQDS
KDSTYSLSSTLTLSKADYEKHKVYACEVTHQGLSSPVTKSFNRGEC;

Heavy chain:
QVQLVQSGAEVKKPGSSVKVSCKASGGTFSSYAISWVRQAPGQGLEWMGRIIPIFG
TANYAQKFQGRVTITADESTSTAYMELSSLRSEDTAVYYCASLASYSSGWEDVFDI
WGQGTMVTVSSASTKGPSVFPLAPSSKSTSGGTAALGCLVKDYFPEPVTVSWNSG
ALTSGVHTFPAVLQSSGLYSLSSVVTVPSSSLGTQTYICNVNHKPSNTKVDKKVEPK
SCDKTHTHHHHHH

For the ACE2 bound Omicron spike ECD protein complex, the Omicron spike ECD protein was incubated with purified peptidase domain of human ACE2 at molar ratios of 1:3.2 (spike trimer to peptidase domain of human ACE2) for 60 min on ice prior to purification by gel filtration chromatography using a Superose6 column (GE Healthcare) pre-equilibrated with TBS buffer (20 mM Tris, pH8.0, 150 mM NaCl). For the JMB2002Fab bound Omicron spike ECD protein complex, the Omicron spike ECD protein was incubated with JMB2002Fab and anti-Fab nanobody at molar ratios of 1:4:5 (spike trimer to JMB2002Fab to anti-Fab nanobody) for 60 min on ice prior to purification by gel filtration chromatography using a Superose6 column (GE Healthcare) pre-equilibrated with HBS buffer (25 mM HEPES, pH 7.5, 150 mM NaCl). For the Omicron spike ECD trimer protein only, the Omicron spike ECD protein was directedly set to purification by gel filtration chromatography using a Superose6 column (GE Healthcare) pre-equilibrated with TBS buffer (20 mM Tris, pH 8.0, 150 mM NaCl) or HBS buffer (25 mM HEPES, pH 7.5, 150 mM NaCl). Fractions containing the complex were pooled and concentrated to 1.5-3 mg/ml.

Cryo-EM sample preparation and data acquisition

For the ACE2 bound Omicron spike ECD protein complex, 3 μL sample purified with TBS buffer at the concentration of 2.5 mg/ml were applied to glow-discharged

UltrAuFoil Holey Gold 300 mesh grids (Au, R1.2/1.3); For the JMB2002Fab bound Omicron spike ECD protein complex, 3 μL sample purified with HBS buffer at the concentration of 2.5 mg/ml were applied to glow-discharged 300 mesh amorphous NiTi foil (Au, 1.2/1.3). Extra samples were blotted with filter paper for 3.0 s and plunge-frozen in liquid ethane using a FEI Vitrobot Mark IV. Cryo-EM micrographs were collected on a 300kV Titan Krios microscope (FEI) equipped with a K3 direct detection camera.

For ECD-ACE2 dataset, 8,194 movies were collected, and for ECD-Fab dataset, 14,740 movies were collected on a Titan Krios equipped with a Gatan K3 direct electron detection device at 300kV with a magnification of 105,000, corresponding to a pixel size 0.824 Å. Image acquisition was performed with EPU Software (FEI Eindhoven, Netherlands). We collected a total of 36 frames accumulating to a total dose of 50 e^{-} $Å^{-2}$ over 2.5 s exposure.

Cryo-EM data processing

For ECD-ACE2 dataset, MotionCor2 was used to perform the frame-based motion-correction algorithm to generate drift-corrected micrograph for further processing and CTFFIND4 provided the estimation of the contrast transfer function (CTF) parameters(*23, 24*). 1,566 aligned micrographs were deleted because of contaminations or bad ice quality. After selection, approximately 1,500 particles were manually picked and two-dimensional (2D) classes were calculated and used as references for automatic picking. A total of 5,071,668 particles were extracted from the cryo-EM micrographs performed with Relion3.0(*25*). All subsequent steps including 2D classification, three-dimensional (3D) classification, 3D refinement, and local refinement were performed using cryoSPARC(*26*), unless stated otherwise. Two rounds of reference-free 2D classification, yielding 854,875 particles after clearance. Three rounds hetero refinement separated out 522,186 particles that resulted to a clearer density of ECD-trimer structure at 2.56 Å global resolution. Two rounds hetero refinement separated out 242,374 particles that resulted to a clearer

density of ACE2 bound ECD-trimer structure at 2.77 Å global resolution. Local refinement focused on the ACE2-RBD or RBD-ACE2-RBD with masks could reconstitute ACE2-RBD and RBD-ACE2-RBD structures at 2.79 Å and 2.57 Å global resolution, respectively. Local resolution estimate was performed with cryoSPARC.

For ECD-Fab dataset, MotionCor2 was used to perform the frame-based motion-correction algorithm to generate drift-corrected micrograph for further processing and CTFFIND4 provided the estimation of the contrast transfer function (CTF) parameters (*23, 24*). Automated particle selection yielded 6,563,376 particles. Two rounds of reference-free 2D classification and three rounds hetero refinement separated out 172,001 particles that resulted to a density of ECD-trimer bound to 1 Fab at 3.18 Å global resolution, 906,588 particles that resulted a density of ECD-trimer bound to 2 Fab at 2.69 Å global resolution, and 434,893 particles that resulted to a density of ECD-trimer bound to 1 Fab at 2.92 Å global resolution. The maps were sharpened by DeepEMhancer (*27*). Local resolution estimate was performed with cryoSPARC. For the structure of RBD-Fab interface, the particles of ECD-trimer bound 2 Fab were performed 3D classification focus on the RBD-Fab interface. Well-defined classes including 483,474 particles were performed local refinement, resulting a density map at 2.47 Å resolution.

Model building

ECD structure from Swiss-model prediction was used as the starting reference model for ECD-trimer building(*28*). Structure of ACE2 derived from PDB entry 7KMB(*29*) was rigid body fit into the density. For the Fab-ECD complex, the structure of the SARS-CoV-2 spike glycoprotein (PDB: 6VXX(*30*)) and the Fab-nanobody-AMPK structure (PDB:7JHH(*21*)) were used as initial model. All models were fitted into the EM density map using UCSF Chimera(*31*) followed by iterative rounds of manual adjustment and automated rebuilding in COOT(*32*) and PHENIX(*33*), respectively. The model was finalized by rebuilding in ISOLDE(*34*) followed by refinement in

PHENIX with torsion-angle restraints to the input model. The final model statistics were validated using Comprehensive validation (cryo-EM) in PHENIX(*33*). All structural figures were prepared using Chimera(*31*), Chimera X(*35*), and PyMOL (Schrödinger, LLC.).

Thermal shift assay (TSA)

The spike ECD and RBD proteins of Omicron and WT SARS-CoV-2 were diluted to 0.5 mg/ml using 200 mM HEPES pH 7.4, 150 mM sodium chloride (final HEPES concentration: 100 mM). Then the diluted proteins were mixed with 10× SYPRO Orange (Thermo Fisher) and incubated for 10 min at room temperature. The reaction was performed in 96-well plates with a final volume of 20 μl. The thermal melting curve were monitored using a LightCycler 480 II Real-Time PCR System (Roche Diagnostics) with a ramp rate of 3.6 °C per minute from 25 °C to 80 °C. The melting peaks were calculated by the LightCycler 480 software provided by Roche Diagnostics. One representative figure from triple experiments is shown.

Measurement of human ACE2 binding to Omicron and WT spike RBD/ECD by biolayer interferometry

The binding of human ACE2 to Omicron and WT spike RBD/ECD were performed using Octet Red96e (Sartorius). The biotinylation of SARS-CoV-2 spike RBD or monomeric human ACE2 protein were carried out by using EZ-Link Sulfo-NHS-LC-Biotin kit (ThermoFisher Scientific, A39257) following the manufacturer’s instruction. SA biosensors (Sartorius, 18-5020) were used to immobilize the biotinylated WT spike RBD protein (Sino Biological, 40589-V08H), Omicron SARS-CoV-2 spike RBD protein (Sino Biological, 40952-V08H121) or monomeric human ACE2 protein. The association of SARS-CoV-2 spike RBD-coated sensors with different concentrations of monomeric human ACE2 were recorded prior to 10 min dissociation in kinetics buffer (0.02% Tween-20 in PBS). The binding of biotinylated monomeric human ACE2 to WT SARS-CoV-2 spike ECD (Sino Biological, 40589-V08B1) or Omicron

SARS-CoV-2 spike ECD (Sino Biological, 40589-V08H26) was also evaluated. Protein A biosensors (Sartorius, 18-5012) were used to capture the dimeric human ACE2 (Sino Biological, 10108-H02H) followed by measuring the association and dissociation with WT or Omicron spike ECD protein. K_D values were calculated with Octet Data Analysis HT 12.0 software using a 1:1 global fit model. Data were plotted using Prism V8.0 software (GraphPad). One representative figure from two independent experiments is shown.

Measurement of anti-SARS-CoV-2 antibody JMB2002 binding to Omicron and WT spike ECD by biolayer interferometry

The interaction of anti-SARS-CoV-2 antibody JMB2002 with Omicron and WT spike ECD was determined using Octet Red96e (Sartorius). SA biosensors (Sartorius, 18-5020) were used to capture the biotinylated WT SARS-CoV-2 spike ECD protein (ACRO, SPN-C82E9) or Omicron SARS-CoV-2 spike ECD protein (ACRO, SPN-C82Ee). The association of SARS-CoV-2 spike protein-coated sensors with different concentrations of anti-SARS-CoV-2 antibody JMB2002 were recorded prior to 10 min dissociation in kinetics buffer (0.02% Tween-20 in PBS). K_D values were calculated with Octet Data Analysis HT 12.0 software using a 1:1 global fit model. Data were plotted using Prism V8.0 software (GraphPad). One representative figure from two independent experiments is shown.

Pseudovirus neutralization assay

In the pseudovirus neutralization assay, serial dilutions of JMB2002 IgG were preincubated with an equal volume of WT, Alpha, Beta, Gamma, Delta and Omicron pseudovirus (2×10^4 $TCID_{50}$/mL) for 1 h at 37°C. Subsequently, HEK293 cells stably expressing hACE2 (Vazyme, DD1401) (2×10^4 cells/well) were seeded in 96-well plates, treated with the corresponding pseudovirus and antibody mixtures, and incubated at 37°C for 48 h. Luciferase activity was measured using Bio-Lite Luciferase Assay

System (Vazyme, DD1201). The neutralization inhibition rate was calculated with the following formula.

$$\text{Inhibition rate } (\%) = \left(1 - \frac{\text{mean intensity of sample} - \text{mean intensity of blank control}}{\text{mean intensity of negative control} - \text{mean intensity of blank control}}\right) X\, 100\%$$

IC_{50} values were calculated by a four-parameter logistic curve fitting approach in Prism V8.0 software (GraphPad). One representative figure from two independent experiments is shown.

AlphaScreen assays for JMB2002 inhibition of ACE2 binding to the Omicron spike ECD trimer

The JMB2002 ability to block ACE2 binding Omicron spike trimer was assessed by luminescence based AlphaScreen technology (PerkinElmer) using a hexahistidine detection kit (*36*). 10 nM ACE2 were attached to streptavidin-coated donor beads, and 10 nM His6-tagged Omicron spike ECD were attached to nickel-chelated acceptor beads. Donor beads contain a photosensitizer that, upon activation at 680 nm, converts ambient oxygen into singlet oxygen. When the acceptor beads are brought into close proximity of the donor beads by ACE2-Omicron spike ECD interaction, energy is transferred from singlet oxygen to thioxene derivatives in the acceptor beads, resulting in light emission at 520 to 620 nm. The binding mixtures, containing the indicated amounts of ACE2 and Omicron spike ECD and streptavidin-coated donor beads (5mg/ml) and Ni-chelate-coated acceptor beads, were incubated in 50 mM Mops (pH 7.4), 100 mM NaCl, and bovine serum albumin (0.1 mg/ml) for 1 to 2 hours before data collection using an EnVision plate reader (PerkinElmer). For the JMB2002 inhibition, increasing concentrations of JMB2002 or Control IgG were added in addition to the ACE2-Omicron spike ECD. The IC_{50} values were derived from curve fitting on the basis of a competitive inhibitor model with GraphPad Prism, using conditions where the concentrations of tagged binding partners were below K_D.

HDX detected by mass spectrometry

HDX-MS were performed as following: 5 µM purified WT or Omicron spike proteins were prepared for HDX reactions at 4 °C. Four-microliter of protein was diluted into 16 µl D2O on exchange buffer (50 mM HEPES, pH 7.5, 50 mM NaCl, 2 mM DTT) and incubated for various HDX time points (e.g., 0, 10, 60, 300 s) at 4 °C and quenched by mixing with 20 µl of ice-cold 3 M gHCL, 1% trifluoroacetic acid. Each quenched sample was immediately injected into the LEAP Pal 3.0 HDX platform. Upon injection, samples were passed through an immobilized pepsin column (2mm × 2cm) at 120 µl min−1 and the digested peptides were captured on a C18 PepMap300 trap column (ThermoFisher) and desalted. Peptides were separated across a 2.1mm × 5cm C18 separating column (1.9 µm Hypersil Gold, ThermoFisher) with a linear gradient of 4% - 40% CH3CN and 0.3% formic acid, over 6 min. Sample handling, protein digestion and peptide separation were conducted at 4°C.

Mass spectrometric data were acquired using a Fusion Orbitrap mass spectrometer (ThermoFisher) with a measured resolving power of 65,000 at m/z 400. HDX analyses were performed in triplicate, with single preparations of each protein ligand complex. The intensity weighted mean m/z centroid value of each peptide envelope was calculated and subsequently converted into a percentage of deuterium incorporation. Statistical significance for the differential HDX data is determined by an unpaired t-test for each time point, a procedure that is integrated into the HDX Workbench software. Corrections for back-exchange were made on the basis of an estimated 70% deuterium recovery, and accounting for the known 80% deuterium content of the deuterium exchange buffer.

Peptides were identified using tandem MS (MS/MS) with a Fusion Orbitrap mass spectrometer (ThermoFisher). Product ion spectra were acquired in data-dependent mode with the top eight most abundant ions selected for the product ion analysis per scan event. The MS/MS data files were submitted to Proteome Discover 2.4 (ThermoFisher) for high confident peptide identification.

The HDX data from all overlapping peptides were consolidated to individual amino acid values using a residue averaging approach. Briefly, for each residue, the deuterium incorporation values and peptide lengths from all overlapping peptides were assembled. A weighting function was applied in which shorter peptides were weighted more heavily and longer peptides were weighted less. Each of the weighted deuterium incorporation values were then averaged to produce a single value for each amino acid. The initial two residues of each peptide, as well as prolines, were omitted from the calculations.

Figure Legends for supplementary figures

Fig. S1. Purification and characterization of the Omicron spike protein complex. (**A**) Gel filtration profile of the Omicron ECD-ACE2 complex, showing a sharp peak, and the SDS gel of the Omicron ECD-ACE2 complex, showing balanced ratios for each subunit. (**B**) Gel filtration profile of the Omicron ECD-Fab complex, showing a sharp peak, and the SDS gel of the Omicron ECD-Fab complex, showing balanced ratios for each subunit. (**C**) Thermal stability shift analysis of the Omicron and WT RBD. (**D**) Thermal stability shift analysis of the Omicron and WT spike trimer .

Fig. S2. Cryo-EM data processing of the Omicron ECD-ACE2 complex. (**A**) A representative cryo-EM micrograph of Omicron ECD-ACE2 complex with 50 nm scale bar included as a size reference. (**B**) Computational processing of cryo-EM data. (**C**) Twenty representative reference-free two-dimensional (2D) cryo-EM class averages reveal the ACE2 density (yellow arrow). (**D**) Local resolution of sub-reconstructions of RBD-ACE2 (left panel) and RBD-ACE2-RBD (right panel). Resolution bar is shown in the middle. (**E**) The FSC curves for the reconstructions. Color scheme: ECD-ACE2, black; RBD-ACE2, red; ECD-trimer, yellow; RBD-ACE2-RBD, blue. The resolution of the reconstructions using the Fourier shell cutoff at 0.143 is shown.

Fig. S3. Cryo-EM analysis of Omicron ECD-trimer. (**A**) High resolution density map of Omicron ECD-trimer after sharpening. Front and top views are shown. The densities for three RBDs are smeared. (**B**) Density map of Omicron ECD-trimer at low resolution of 4.5 Å without sharpening. (**C**) Density map of Omicron ECD-trimer at low resolution 6.5 Å without sharpening. The blob densities according to RBDs are shown in all-down conformation. Front and top views are shown. Three ECDs are colored in purple, green, and salmon.

Fig. S4 Comparative HDX analysis on WT vs Omicron Spike protein. (**A**) Single amide consolidated HDX-MS profiling of *apo* WT Spike vs apo Omicron full-length Spike protein. The y- and x-axes illustrate the differential D% ($HDX_{Omicron}$ minus HDX_{WT}) and residue numbers of aligned WT and Omicron Spike protein, respectively. (**B**) Differential HDX data consolidated from Fig. S1 and Fig.S2 are mapped to Full-length Omicron spike conformer that displays an up-oriented RBD, according to the differential HDX dynamics key shown below. Regions that show increased HDX activity (more disordered) are colored orange/red; regions that show decreased HDX activity (more stable) are colored blue.

Fig. S5 The mutation-induced conformations change of Omicron RBD comparing with WT RBD. (A) Comparison RBD-RBD interface between Omicron RBD and WT RBD (PDB:6LZG). Left panel, electrostatic potential maps between Omicron RBD and WT RBD, positive and negative charged regions are in blue and red, respectively. Right panel, superposition of the Omicron RBD and WT RBD (PDB: 6LZG). **(B)** Comparison Omicron RBD with a WT RBD that complexed with lipid acid (PDB: 6ZB4). Left panel, electrostatic potential maps between Omicron RBD and WT RBD. Right panel, superposition of the Omicron RBD and WT RBD (PDB: 6ZB4).

Fig. S6 Neutralization of WT, Alpha, Beta, Gamma, and Delta pseudo-virus by JMB2002. Delta IC_{50} values were calculated by Prism V8.0 software using a four-parameter logistic curve fitting approach.

Fig. S7. Cryo-EM data processing of the Omicron ECD-Fab complex. (**A**) Flow chart of cryo-EM analysis. (**B**) Representative cryo-EM image (scale bar, 50 nm) from 14,740 movies. (**C**) Representative 2D averages (scale bar, 5 nm). (D-F) The EM maps of Omicron ECD trimer bound to 1 Fab (F), 2 Fab (D), 3 Fab (E) and (G)

the focus refinement density map of the binding interfaces between one RBD and Fab complex.

Fig. S8. Cryo-EM maps and model of the Omicron ECD-Fab complexes.
(**A-D**) The map and fitted model and the 'Gold-standard' FSC curves of the global complexes of the Omicron ECD spike bound to 1 Fab (A), 2 Fab (B), 3 Fab (C) and the one RBD-Fab interface (D)

Fig. S9. Structural comparisons of the ACE2-bound and Fab-bound SARS-CoV-2 Omicron Spike protein. (**A-B**) Superpositions of the ACE2-bound and Fab-bound Omicron Spike trimer. The different protomers are colored. The compared models showed with a front view (A) and a top view (B). (**C**) Superpositions shows ACE2 and the Fab do not share binding sites of S protein RBD.

Fig. S10. Different states of the Omicron ECD-Fab complexes. (**A**) The cryo-EM density map and model of 3 Fab-bound ECD-Fab complex showed in front view and top view. (**B**) The cryo-EM density map and model of 1 Fab-bound ECD-Fab complex showed in front view and top view.

Fig. S1

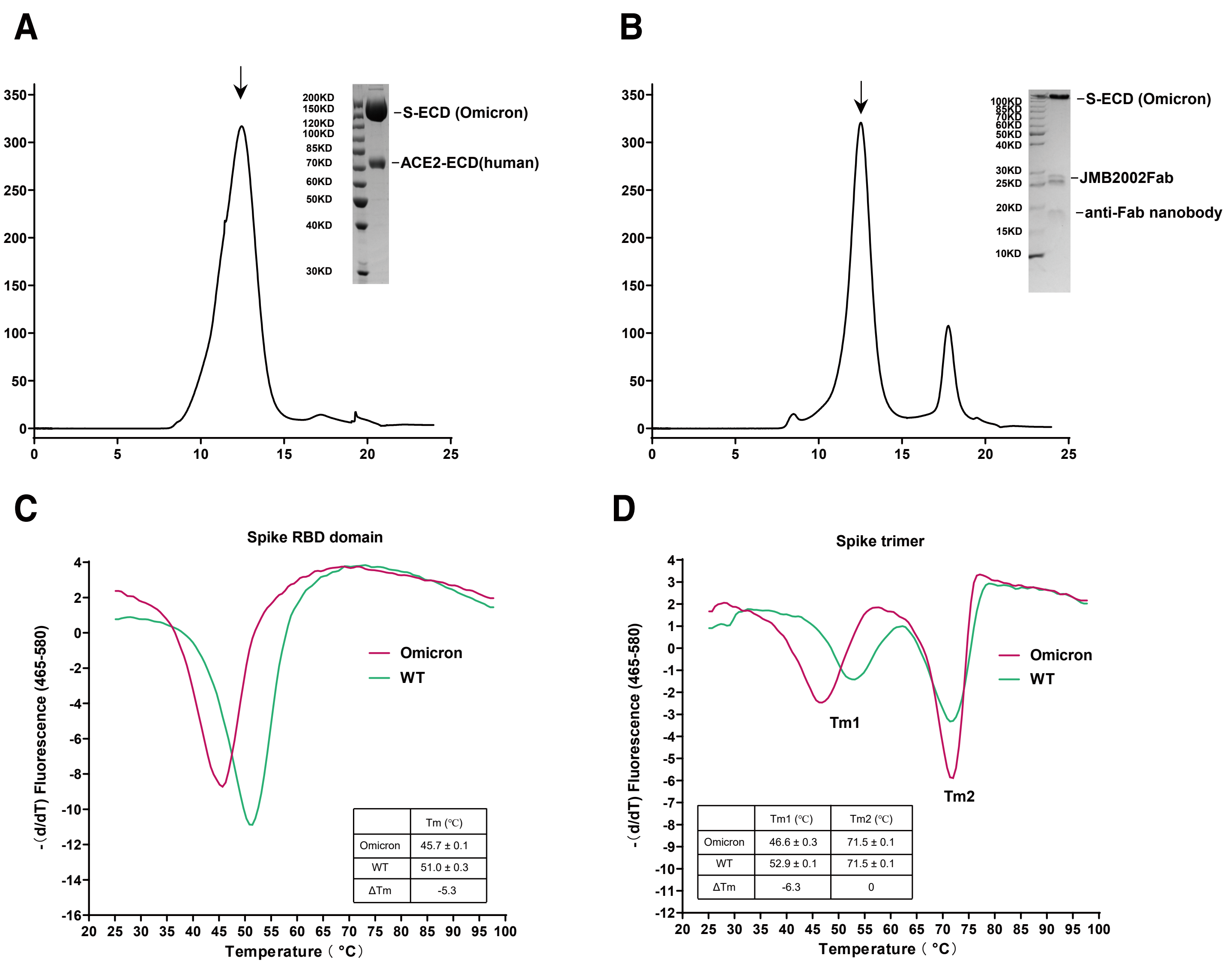
A
B
C
D
200KD
150KD
120KD
100KD
85KD
70KD
60KD
50KD
40KD
30KD
S-ECD (Omicron)
ACE2-ECD(human)
100KD
85KD
70KD
60KD
50KD
40KD
30KD
25KD
20KD
15KD
10KD
S-ECD (Omicron)
JMB2002Fab
anti-Fab nanobody
Spike RBD domain
-(d/dT) Fluorescence (465-580)
Temperature (°C)
Omicron
WT
Tm (℃)
Omicron 45.7 ± 0.1
WT 51.0 ± 0.3
ΔTm -5.3
Spike trimer
Tm1
Tm2
Tm1 (℃)
Tm2 (℃)
Omicron 46.6 ± 0.3 71.5 ± 0.1
WT 52.9 ± 0.1 71.5 ± 0.1
ΔTm -6.3 0

Fig. S2

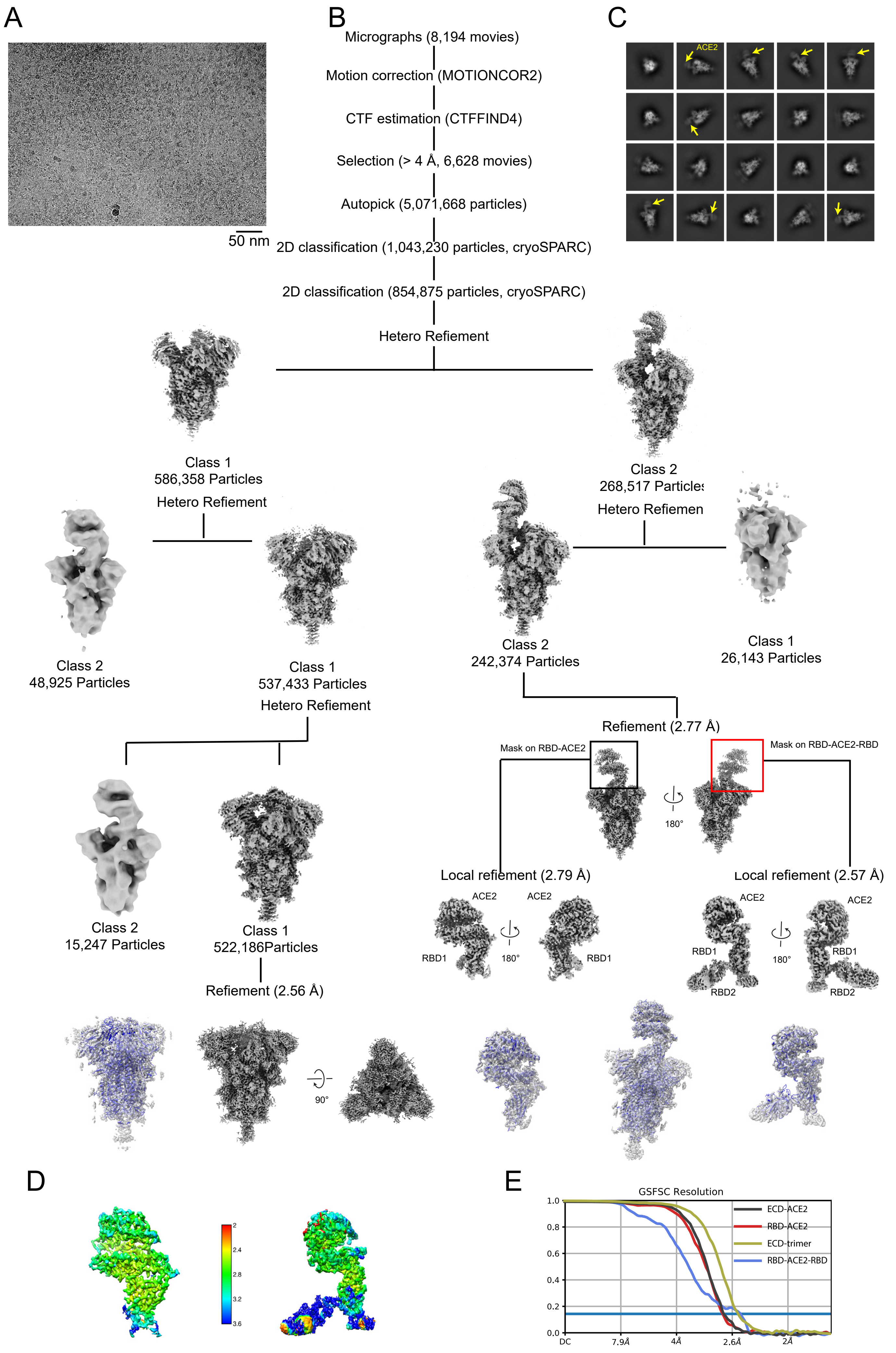
A
50 nm
B
Micrographs (8,194 movies)
Motion correction (MOTIONCOR2)
CTF estimation (CTFFIND4)
Selection (> 4 Å, 6,628 movies)
Autopick (5,071,668 particles)
2D classification (1,043,230 particles, cryoSPARC)
2D classification (854,875 particles, cryoSPARC)
Hetero Refiement
C
ACE2
Class 1
586,358 Particles
Hetero Refiement
Class 2
268,517 Particles
Hetero Refiemen
Class 2
48,925 Particles
Class 1
537,433 Particles
Hetero Refiement
Class 2
242,374 Particles
Class 1
26,143 Particles
Refiement (2.77 Å)
Mask on RBD-ACE2
Mask on RBD-ACE2-RBD
180°
Local refiement (2.79 Å)
Local refiement (2.57 Å)
ACE2
ACE2
RBD1
180°
RBD1
ACE2
ACE2
RBD1
180°
RBD1
RBD2
RBD2
Class 2
15,247 Particles
Class 1
522,186Particles
Refiement (2.56 Å)
90°
D
2
2.4
2.8
3.2
3.6
E
GSFSC Resolution
1.0
0.8
0.6
0.4
0.2
0.0
DC
7.9Å
4Å
2.6Å
2Å
ECD-ACE2
RBD-ACE2
ECD-trimer
RBD-ACE2-RBD

Fig. S3

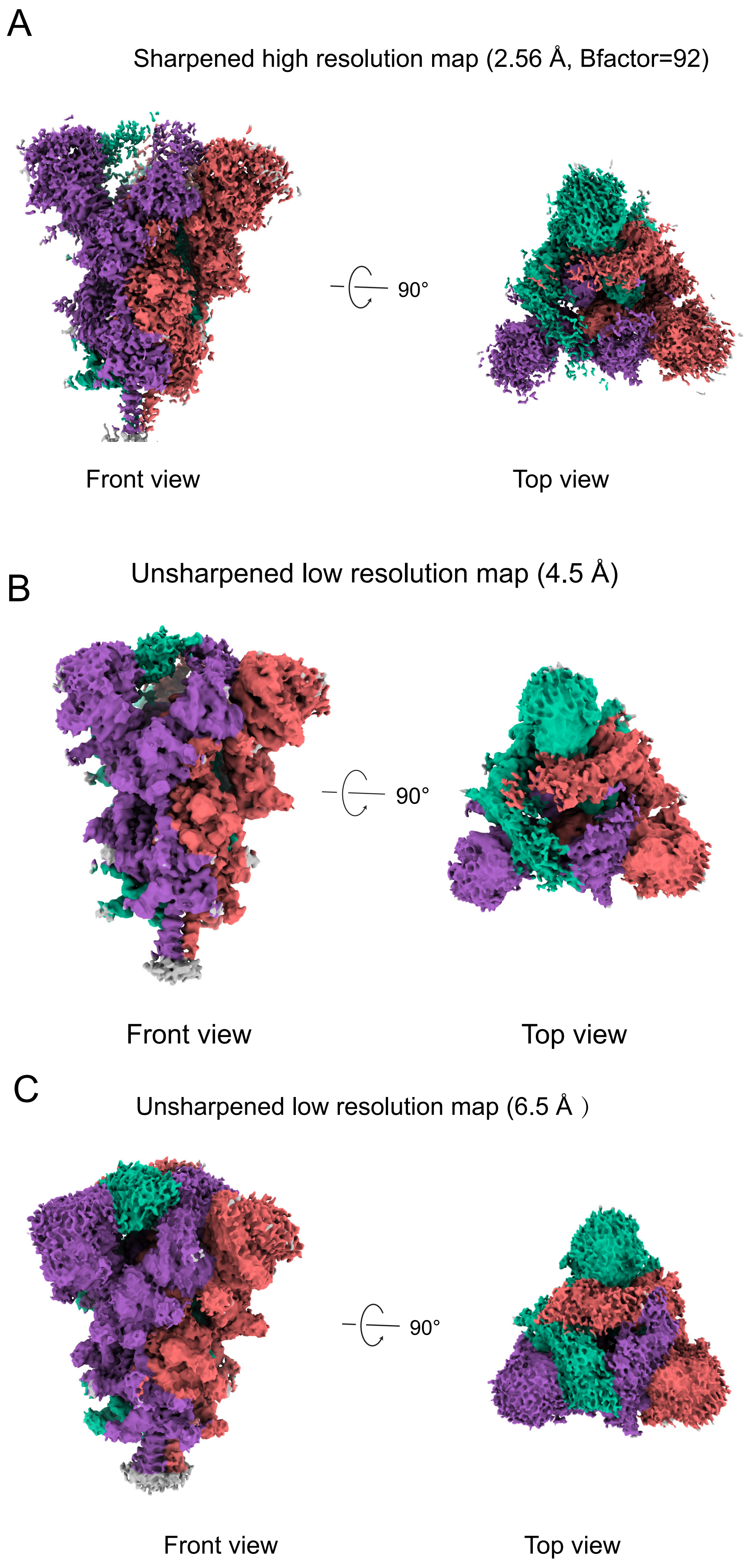
A
Sharpened high resolution map (2.56 Å, Bfactor=92)
90°
Front view
Top view
B
Unsharpened low resolution map (4.5 Å)
90°
Front view
Top view
C
Unsharpened low resolution map (6.5 Å)
90°
Front view
Top view

Fig. S4

A

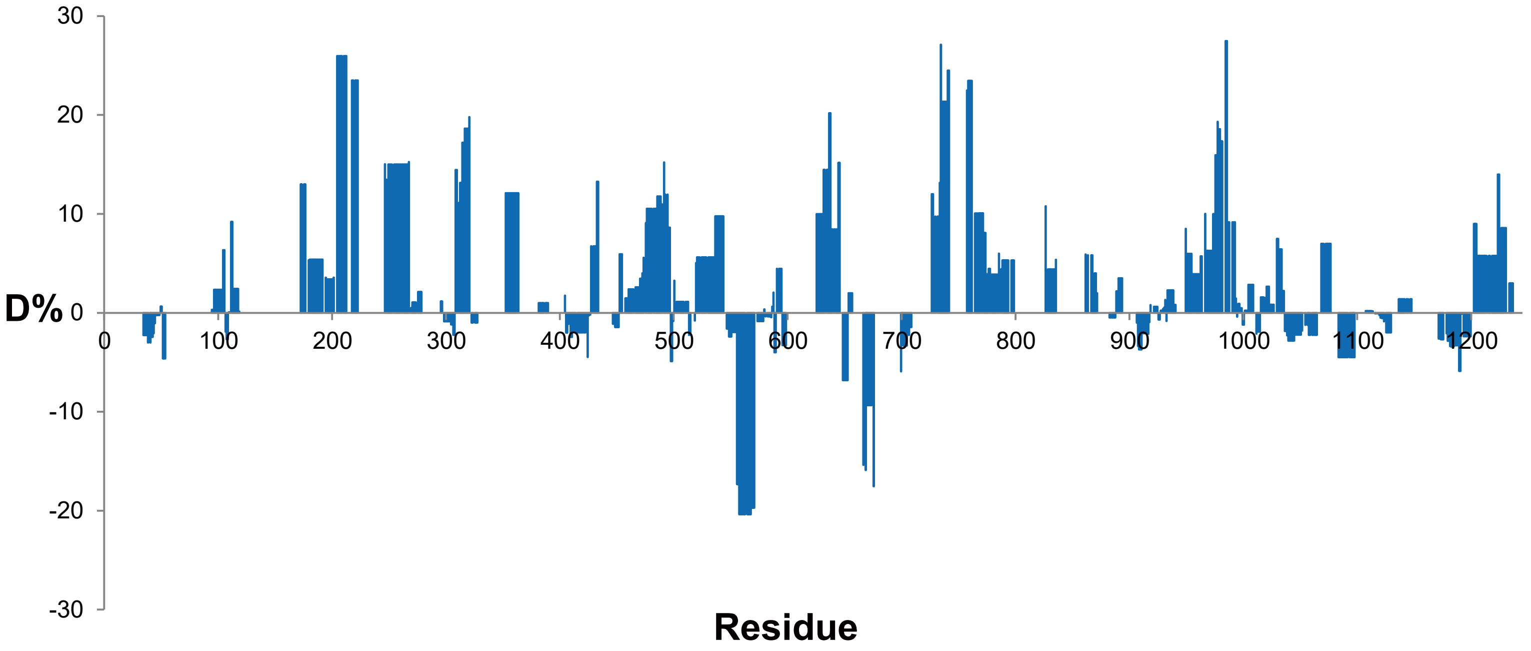
30
20
10
D% 0
-10
-20
-30
0
100
200
300
400
500
600
700
800
900
1000
1100
1200
Residue

B

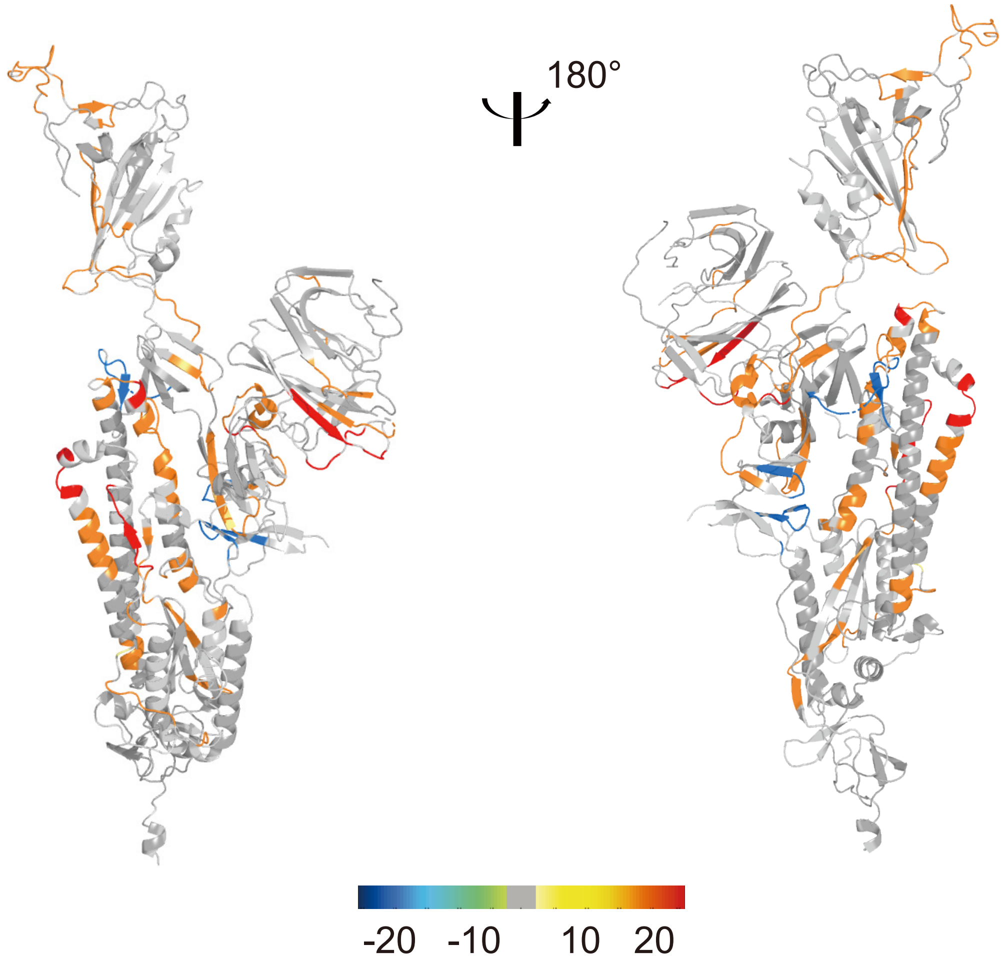
180°
-20
-10
10
20

Fig. S5

A

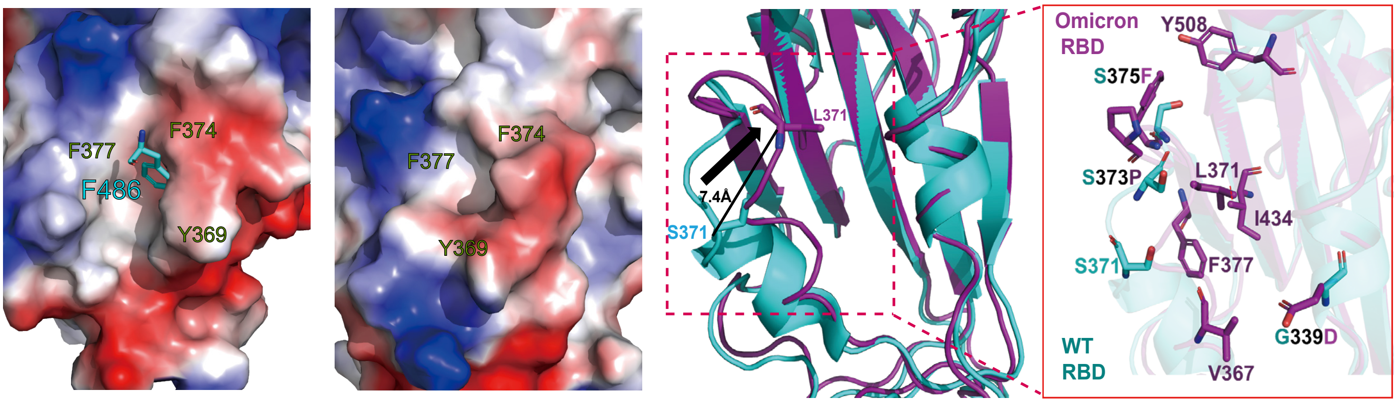

Omicron RBD up

WT RBD (crystal)

B

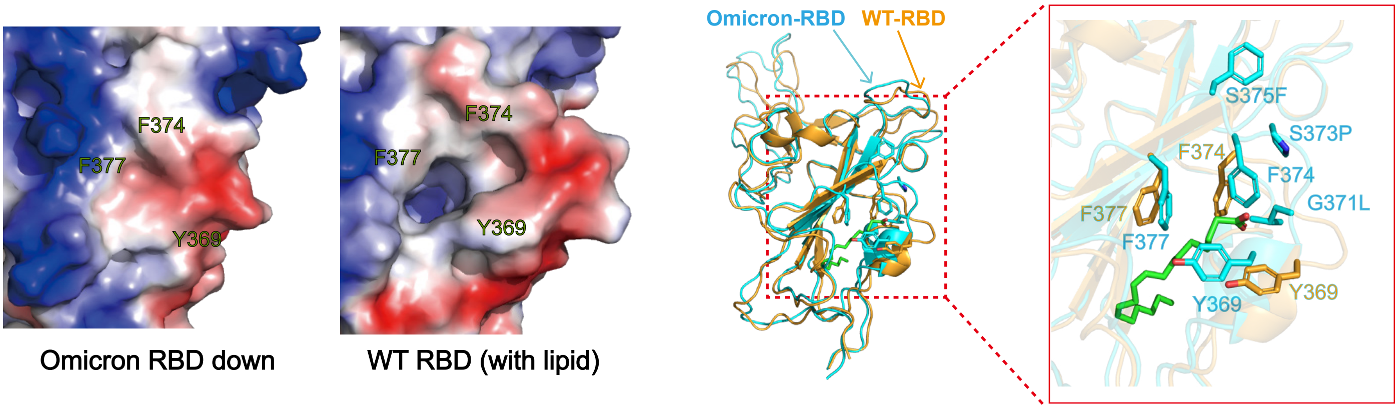

Omicron RBD down

WT RBD (with lipid)

Fig. S6

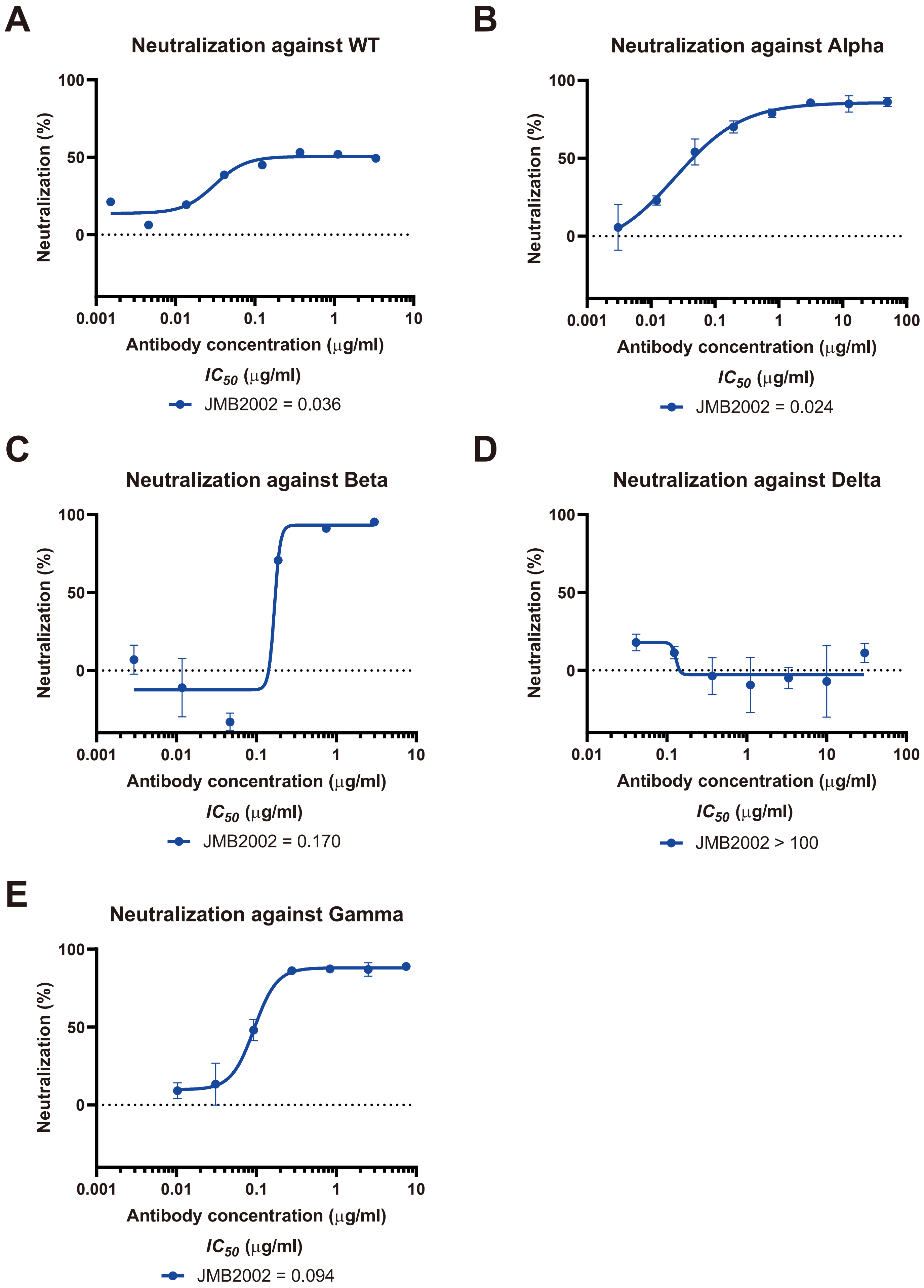
A
Neutralization against WT
Neutralization (%)
Antibody concentration (μg/ml)
IC_{50} (μg/ml)
JMB2002 = 0.036
B
Neutralization against Alpha
Neutralization (%)
Antibody concentration (μg/ml)
IC_{50} (μg/ml)
JMB2002 = 0.024
C
Neutralization against Beta
Neutralization (%)
Antibody concentration (μg/ml)
IC_{50} (μg/ml)
JMB2002 = 0.170
D
Neutralization against Delta
Neutralization (%)
Antibody concentration (μg/ml)
IC_{50} (μg/ml)
JMB2002 > 100
E
Neutralization against Gamma
Neutralization (%)
Antibody concentration (μg/ml)
IC_{50} (μg/ml)
JMB2002 = 0.094

Fig. S7

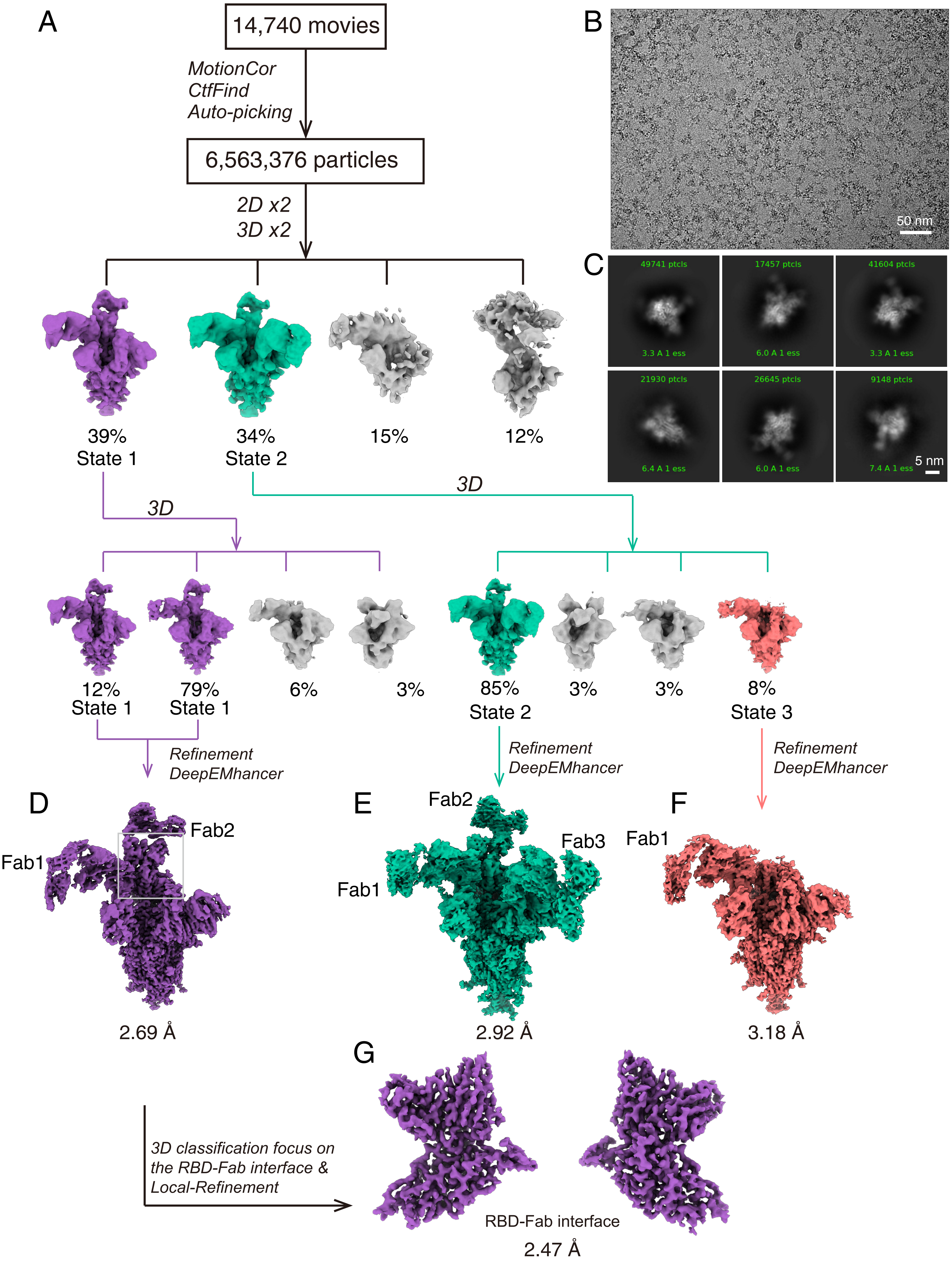
A
14,740 movies
MotionCor
CtfFind
Auto-picking
6,563,376 particles
2D x2
3D x2
39%
State 1
34%
State 2
15%
12%
3D
3D
12%
State 1
79%
State 1
6%
3%
85%
State 2
3%
3%
8%
State 3
Refinement
DeepEMhancer
Refinement
DeepEMhancer
Refinement
DeepEMhancer
B
50 nm
C
49741 ptcls
17457 ptcls
41604 ptcls
3.3 A 1 ess
6.0 A 1 ess
3.3 A 1 ess
21930 ptcls
26645 ptcls
9148 ptcls
6.4 A 1 ess
6.0 A 1 ess
7.4 A 1 ess
5 nm
D
Fab1
Fab2
2.69 Å
E
Fab2
Fab3
Fab1
2.92 Å
F
Fab1
3.18 Å
G
3D classification focus on
the RBD-Fab interface &
Local-Refinement
RBD-Fab interface
2.47 Å

Fig. S8

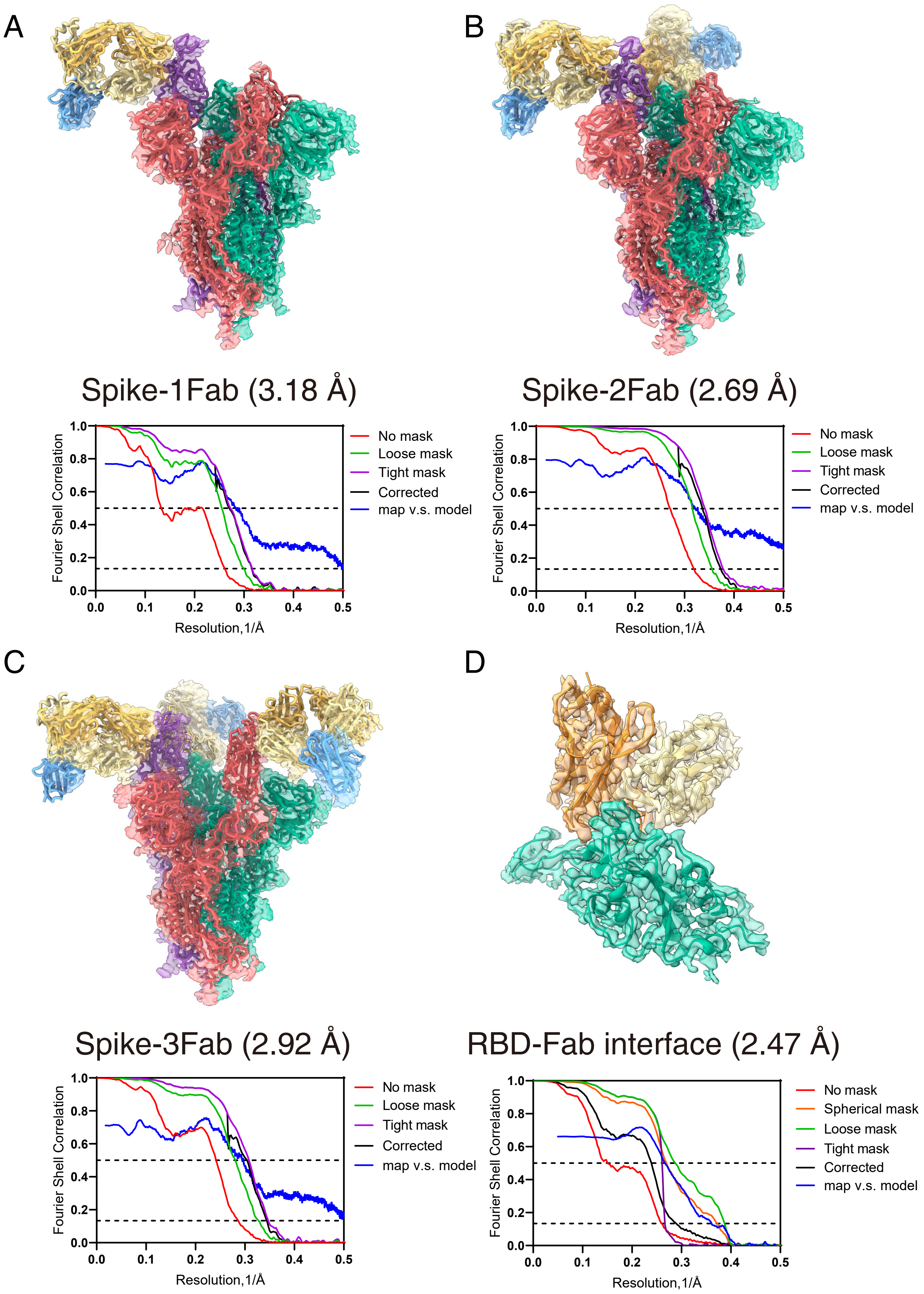
A
B
Spike-1Fab (3.18 Å)
Spike-2Fab (2.69 Å)
Fourier Shell Correlation
Resolution,1/Å
No mask
Loose mask
Tight mask
Corrected
map v.s. model
C
D
Spike-3Fab (2.92 Å)
RBD-Fab interface (2.47 Å)
Spherical mask

Fig. S9

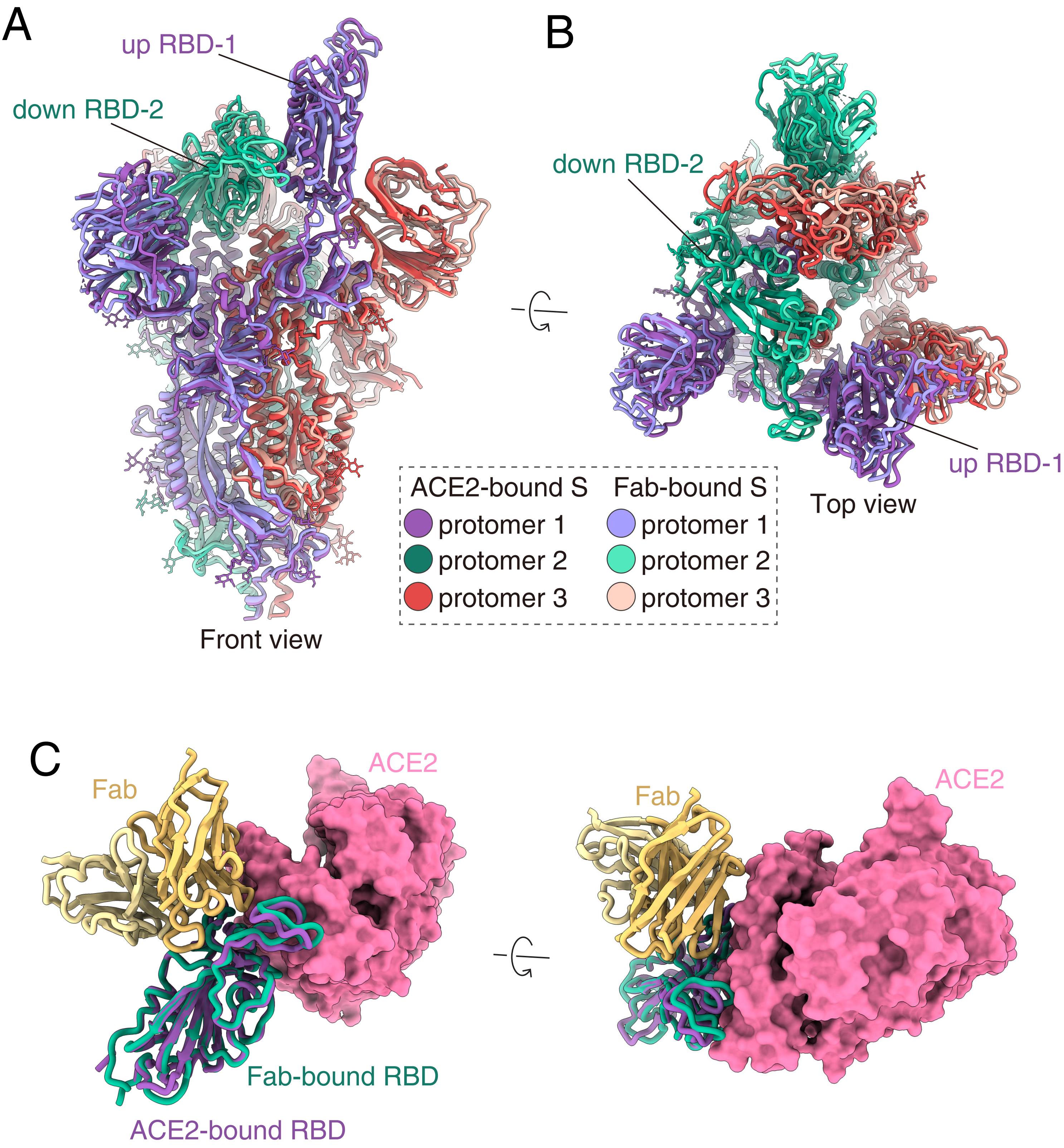
A
up RBD-1
down RBD-2
Front view
B
down RBD-2
up RBD-1
Top view
ACE2-bound S
protomer 1
protomer 2
protomer 3
Fab-bound S
protomer 1
protomer 2
protomer 3
C
Fab
ACE2
Fab-bound RBD
ACE2-bound RBD
Fab
ACE2

Fig. S10

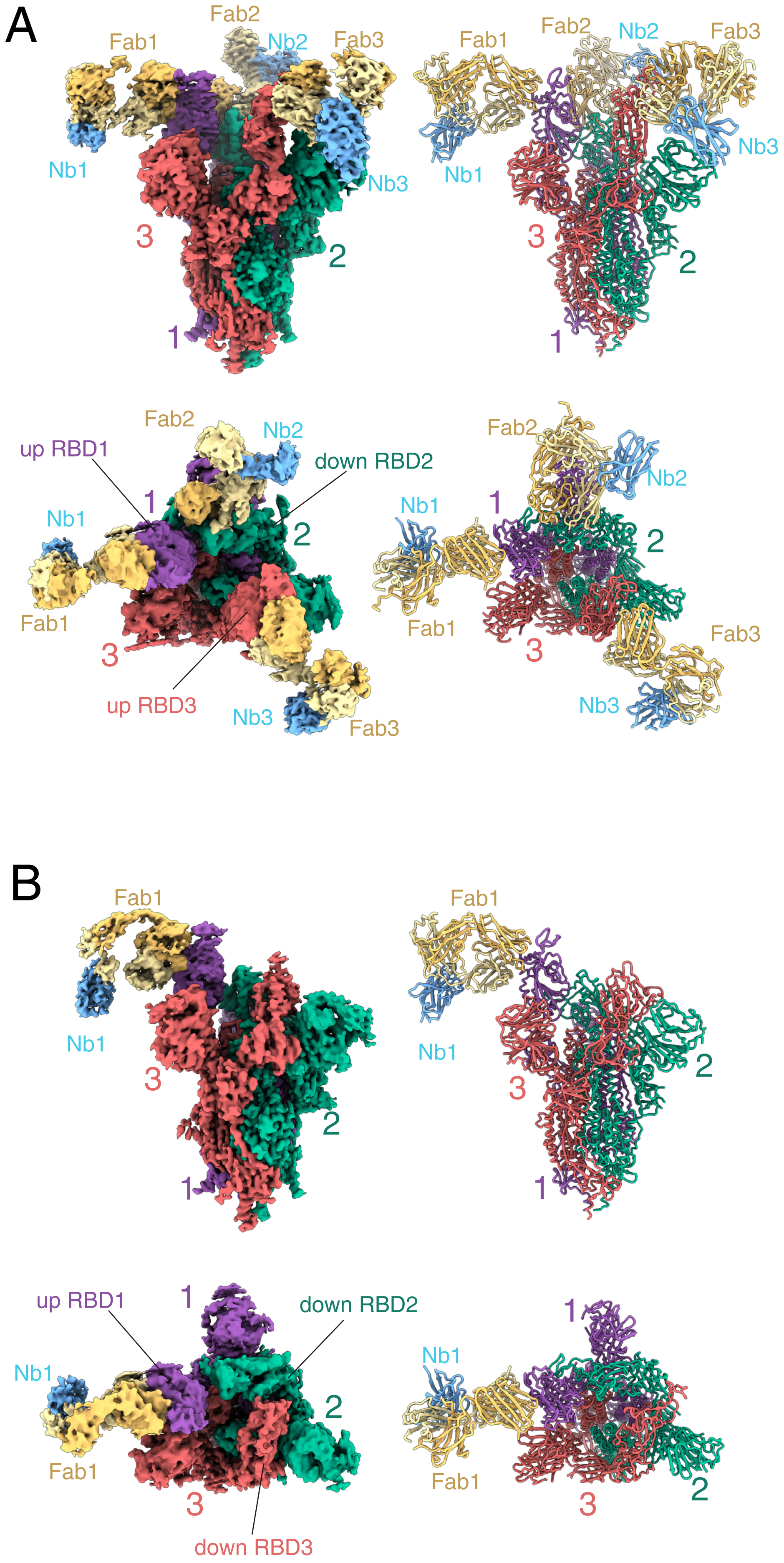
A
Fab1
Fab2
Nb2
Fab3
Nb1
Nb3
3
2
1
up RBD1
down RBD2
up RBD3
B
down RBD3

Table S1 Cryo-EM data collection, refinement and validation statistics

	Omicron Spike-ACE2 complex				Omicron Spike-Fab complex			
**Data collection and processing**								
Magnification	105,000				105,000			
Voltage (kV)	300				300			
Electron exposure ($e^{-}/Å^2$)	50				50			
Defocus range (μm)	-0.5 ~ -2.5				-0.5 ~ -2.5			
Pixel size (Å)	0.824				0.824			
Symmetry imposed	C1				C1			
Initial particle projections (no.)	5,071,668				6,563,376			
Sub-maps	Map 1 Spike-trimer	Map 2 Spike-ACE2	Map 3 RBD-ACE2	Map 4 RBD-ACE2-RBD	Map 1 Spike-1Fab	Map 2 Spike-2Fab	Map 3 Spike-3Fab	Map 4 RBD-Fab interface
Final particle projections (no.)	522,186	242,374	242,374	242,374	172,001	906,588	434,893	483,474
Map resolution (Å)	2.56	2.77	2.79	2.56	3.18	2.69	2.92	2.47
Map resolution range (Å)	2.6-5.5	2.8-6.2	2.8-3.2	2.6-5.5	2.5-6.0	2.5-6.0	2.5-6.0	2.0-4.5
FSC threshold	0.143	0.143	0.143	0.143	0.143	0.143	0.143	0.143
**Model Refinement**								
Refinement package	PHENIX-1.17.1-3660							
Real or reciprocal space	Real space							
Model-Map CC (mask)	0.72	0.73	0.86	0.72	0.69	0.72	0.65	0.75
Model resolution (Å)	2.98	3.19	2.84	3.13	3.18	2.69	2.92	2.47
FSC threshold	0.5	0.5	0.5	0.5	0.5	0.5	0.5	0.5
B factors ($Å^2$, mean value)								
Protein residues	77.42	96.72	50.26	66.21	109.37	77.67	95.21	36.61
Ligands	67.18	30.65	58.28	84.75	116.35	68.81	70.89	N/A
**Model composition**								
Non-hydrogen atoms	25,077	30,450	6,491	8,066	28,092	32,162	36,358	3,271
Protein residues	3,141	3,774	793	982	3,654	4,188	4,746	428
R.m.s. deviations								
Bond lengths (Å)	0.003	0.003	0.005	0.003	0.004	0.006	0.004	0.002
Bond angles (°)	0.775	0.738	0.734	0.678	0.971	1.239	0.999	0.535
**Validation**								
MolProbity score	1.54	1.5	1.35	1.30	1.42	1.42	1.60	1.27
Clashscore	4.05	3.37	3.78	2.47	4.53	4.12	5.83	2.99
Rotamer outliers (%)	0.40	0.18	0.29	0	0.14	0.18	0.13	0.58
Ramachandran plot								
Favored (%)	94.96	94.61	96.96	96.07	96.84	96.51	95.88	96.92
Allowed (%)	4.94	5.39	3.04	3.93	3.16	3.49	4.12	3.08
Disallowed (%)	0.10	0	0	0	0	0	0	0
**Data availability**								
EMDB entry	32679	32680	32681	32682	32683	32684	32685	32736
PDB entry	7WP9	7WPA	7WPB	7WPC	7WPD	7WPE	7WPF	7WRV

Table S3

A summary of the comparison between our Omicron ACE2-RBD structure and selected ACE2-RBD structures.

Our structure Compared with		6M0J	6LZG	6M17	7A94	7KMB	7DF4
Method		X-ray	X-ray	Cryo-EM	Cryo-EM	Cryo-EM	Cryo-EM
Resolution (Å)		2.45	2.5	2.9	3.4	3.39	3.8
RMSD (Å)	Overall	0.49	0.44	0.60	0.87	1.18	1.34
	ACE2	0.49	0.44	0.60	0.83	1.18	1.34
	RBD	0.40	0.34	0.50	0.64	0.66	0.96
Reference (PMID)		32225176	32275855	32132184	32942285	33271067	33277323

References and Notes

1. J. Ai, H. Zhang, Y. Zhang, K. Lin, Y. Zhang, J. Wu, Y. Wan, Y. Huang, J. Song, Z. Fu, H. Wang, J. Guo, N. Jiang, M. Fan, Y. Zhou, Y. Zhao, Q. Zhang, Q. Liu, J. Lv, P. Li, C. Qiu, W. Zhang, Omicron variant showed lower neutralizing sensitivity than other SARS-CoV-2 variants to immune sera elicited by vaccines after boost. *Emerg. Microbes Infect.* **11**, 337–343 (2022). Medline

2. M. Yuan, D. Huang, C. D. Lee, N. C. Wu, A. M. Jackson, X. Zhu, H. Liu, L. Peng, M. J. van Gils, R. W. Sanders, D. R. Burton, S. M. Reincke, H. Prüss, J. Kreye, D. Nemazee, A. B. Ward, I. A. Wilson, Structural and functional ramifications of antigenic drift in recent SARS-CoV-2 variants. *Science* **373**, 818–823 (2021). doi:10.1126/science.abh1139 Medline

3. Y. Cai, J. Zhang, T. Xiao, C. L. Lavine, S. Rawson, H. Peng, H. Zhu, K. Anand, P. Tong, A. Gautam, S. Lu, S. M. Sterling, R. M. Walsh Jr., S. Rits-Volloch, J. Lu, D. R. Wesemann, W. Yang, M. S. Seaman, B. Chen, Structural basis for enhanced infectivity and immune evasion of SARS-CoV-2 variants. *Science* **373**, 642–648 (2021). doi:10.1126/science.abi9745 Medline

4. R. Earnest, R. Uddin, N. Matluk, N. Renzette, K. J. Siddle, C. Loreth, G. Adams, C. H. Tomkins-Tinch, M. E. Petrone, J. E. Rothman, M. I. Breban, R. T. Koch, K. Billig, J. R. Fauver, C. B. F. Vogels, S. Turbett, K. Bilguvar, B. De Kumar, M. L. Landry, D. R. Peaper, K. Kelly, G. Omerza, H. Grieser, S. Meak, J. Martha, H. H. Dewey, S. Kales, D. Berenzy, K. Carpenter-Azevedo, E. King, R. C. Huard, S. C. Smole, C. M. Brown, T. Fink, A. S. Lang, G. R. Gallagher, P. C. Sabeti, S. Gabriel, B. L. MacInnis, New England Variant Investigation Team, R. Tewhey, M. D. Adams, D. J. Park, J. E. Lemieux, N. D. Grubaugh, Comparative transmissibility of SARS-CoV-2 variants Delta and Alpha in New England, USA. medRxiv 2021.10.06.21264641 [Preprint] (2021). https://doi.org/10.1101/2021.10.06.21264641.

5. K. B. Pouwels, E. Pritchard, P. C. Matthews, N. Stoesser, D. W. Eyre, K.-D. Vihta, T. House, J. Hay, J. I. Bell, J. N. Newton, J. Farrar, D. Crook, D. Cook, E. Rourke, R. Studley, T. E. A. Peto, I. Diamond, A. S. Walker, Effect of Delta variant on viral burden and vaccine effectiveness against new SARS-CoV-2 infections in the UK. *Nat. Med.* **27**, 2127–2135 (2021). doi:10.1038/s41591-021-01548-7 Medline

6. Y. Cao, J. Wang, F. Jian, T. Xiao, W. Song, A. Yisimayi, W. Huang, Q. Li, P. Wang, R. An, J. Wang, Y. Wang, X. Niu, S. Yang, H. Liang, H. Sun, T. Li, Y. Yu, Q. Cui, S. Liu, X. Yang, S. Du, Z. Zhang, X. Hao, F. Shao, R. Jin, X. Wang, J. Xiao, Y. Wang, X. S. Xie, Omicron escapes the majority of existing SARS-CoV-2 neutralizing antibodies. *Nature* (2021). doi:10.1038/s41586-021-04385-3 Medline

7. D. Planas, N. Saunders, P. Maes, F. Guivel-Benhassine, C. Planchais, J. Buchrieser, W.-H. Bolland, F. Porrot, I. Staropoli, F. Lemoine, H. Péré, D. Veyer, J.

Puech, J. Rodary, G. Baele, S. Dellicour, J. Raymenants, S. Gorissen, C. Geenen, B. Vanmechelen, T. Wawina-Bokalanga, J. Martí-Carreras, L. Cuypers, A. Sève, L. Hocqueloux, T. Prazuck, F. Rey, E. Simon-Loriere, T. Bruel, H. Mouquet, E. André, O. Schwartz, Considerable escape of SARS-CoV-2 Omicron to antibody neutralization. *Nature* (2021). doi:10.1038/s41586-021-04389-z Medline

8. L. Liu, S. Iketani, Y. Guo, J. F.-W. Chan, M. Wang, L. Liu, Y. Luo, H. Chu, Y. Huang, M. S. Nair, J. Yu, K. K.-H. Chik, T. T.-T. Yuen, C. Yoon, K. K.-W. To, H. Chen, M. T. Yin, M. E. Sobieszczyk, Y. Huang, H. H. Wang, Z. Sheng, K.-Y. Yuen, D. D. Ho, Striking antibody evasion manifested by the Omicron variant of SARS-CoV-2. *Nature* (2021). doi:10.1038/s41586-021-04388-0 Medline

9. D. Wrapp, N. Wang, K. S. Corbett, J. A. Goldsmith, C.-L. Hsieh, O. Abiona, B. S. Graham, J. S. McLellan, Cryo-EM structure of the 2019-nCoV spike in the prefusion conformation. *Science* **367**, 1260–1263 (2020). doi:10.1126/science.abb2507 Medline

10. C. L. Hsieh, J. A. Goldsmith, J. M. Schaub, A. M. DiVenere, H.-C. Kuo, K. Javanmardi, K. C. Le, D. Wrapp, A. G. Lee, Y. Liu, C.-W. Chou, P. O. Byrne, C. K. Hjorth, N. V. Johnson, J. Ludes-Meyers, A. W. Nguyen, J. Park, N. Wang, D. Amengor, J. J. Lavinder, G. C. Ippolito, J. A. Maynard, I. J. Finkelstein, J. S. McLellan, Structure-based design of prefusion-stabilized SARS-CoV-2 spikes. *Science* **369**, 1501–1505 (2020). doi:10.1126/science.abd0826 Medline

11. E. Cameroni, J. E. Bowen, L. E. Rosen, C. Saliba, S. K. Zepeda, K. Culap, D. Pinto, L. A. VanBlargan, A. De Marco, J. di Iulio, F. Zatta, H. Kaiser, J. Noack, N. Farhat, N. Czudnochowski, C. Havenar-Daughton, K. R. Sprouse, J. R. Dillen, A. E. Powell, A. Chen, C. Maher, L. Yin, D. Sun, L. Soriaga, J. Bassi, C. Silacci-Fregni, C. Gustafsson, N. M. Franko, J. Logue, N. T. Iqbal, I. Mazzitelli, J. Geffner, R. Grifantini, H. Chu, A. Gori, A. Riva, O. Giannini, A. Ceschi, P. Ferrari, P. E. Cippà, A. Franzetti-Pellanda, C. Garzoni, P. J. Halfmann, Y. Kawaoka, C. Hebner, L. A. Purcell, L. Piccoli, M. S. Pizzuto, A. C. Walls, M. S. Diamond, A. Telenti, H. W. Virgin, A. Lanzavecchia, G. Snell, D. Veesler, D. Corti, Broadly neutralizing antibodies overcome SARS-CoV-2 Omicron antigenic shift. *Nature* (2021). doi:10.1038/s41586-021-04386-2 Medline

12. J. Juraszek, L. Rutten, S. Blokland, P. Bouchier, R. Voorzaat, T. Ritschel, M. J. G. Bakkers, L. L. R. Renault, J. P. M. Langedijk, Stabilizing the closed SARS-CoV-2 spike trimer. *Nat. Commun.* **12**, 244 (2021). doi:10.1038/s41467-020-20321-x Medline

13. X. Chi, R. Yan, J. Zhang, G. Zhang, Y. Zhang, M. Hao, Z. Zhang, P. Fan, Y. Dong, Y. Yang, Z. Chen, Y. Guo, J. Zhang, Y. Li, X. Song, Y. Chen, L. Xia, L. Fu, L. Hou, J. Xu, C. Yu, J. Li, Q. Zhou, W. Chen, A neutralizing human

antibody binds to the N-terminal domain of the Spike protein of SARS-CoV-2. *Science* **369**, 650–655 (2020). doi:10.1126/science.abc6952 Medline

14. J. Hansen, A. Baum, K. E. Pascal, V. Russo, S. Giordano, E. Wloga, B. O. Fulton, Y. Yan, K. Koon, K. Patel, K. M. Chung, A. Hermann, E. Ullman, J. Cruz, A. Rafique, T. Huang, J. Fairhurst, C. Libertiny, M. Malbec, W. Y. Lee, R. Welsh, G. Farr, S. Pennington, D. Deshpande, J. Cheng, A. Watty, P. Bouffard, R. Babb, N. Levenkova, C. Chen, B. Zhang, A. Romero Hernandez, K. Saotome, Y. Zhou, M. Franklin, S. Sivapalasingam, D. C. Lye, S. Weston, J. Logue, R. Haupt, M. Frieman, G. Chen, W. Olson, A. J. Murphy, N. Stahl, G. D. Yancopoulos, C. A. Kyratsous, Studies in humanized mice and convalescent humans yield a SARS-CoV-2 antibody cocktail. *Science* **369**, 1010–1014 (2020). doi:10.1126/science.abd0827 Medline

15. B. E. Jones, P. L. Brown-Augsburger, K. S. Corbett, K. Westendorf, J. Davies, T. P. Cujec, C. M. Wiethoff, J. L. Blackbourne, B. A. Heinz, D. Foster, R. E. Higgs, D. Balasubramaniam, L. Wang, Y. Zhang, E. S. Yang, R. Bidshahri, L. Kraft, Y. Hwang, S. Žentelis, K. R. Jepson, R. Goya, M. A. Smith, D. W. Collins, S. J. Hinshaw, S. A. Tycho, D. Pellacani, P. Xiang, K. Muthuraman, S. Sobhanifar, M. H. Piper, F. J. Triana, J. Hendle, A. Pustilnik, A. C. Adams, S. J. Berens, R. S. Baric, D. R. Martinez, R. W. Cross, T. W. Geisbert, V. Borisevich, O. Abiona, H. M. Belli, M. de Vries, A. Mohamed, M. Dittmann, M. I. Samanovic, M. J. Mulligan, J. A. Goldsmith, C.-L. Hsieh, N. V. Johnson, D. Wrapp, J. S. McLellan, B. C. Barnhart, B. S. Graham, J. R. Mascola, C. L. Hansen, E. Falconer, The neutralizing antibody, LY-CoV555, protects against SARS-CoV-2 infection in nonhuman primates. *Sci. Transl. Med.* **13**, eabf1906 (2021). doi:10.1126/scitranslmed.abf1906 Medline

16. D. Pinto, Y.-J. Park, M. Beltramello, A. C. Walls, M. A. Tortorici, S. Bianchi, S. Jaconi, K. Culap, F. Zatta, A. De Marco, A. Peter, B. Guarino, R. Spreafico, E. Cameroni, J. B. Case, R. E. Chen, C. Havenar-Daughton, G. Snell, A. Telenti, H. W. Virgin, A. Lanzavecchia, M. S. Diamond, K. Fink, D. Veesler, D. Corti, Cross-neutralization of SARS-CoV-2 by a human monoclonal SARS-CoV antibody. *Nature* **583**, 290–295 (2020). doi:10.1038/s41586-020-2349-y Medline

17. Q. Wang, Y. Zhang, L. Wu, S. Niu, C. Song, Z. Zhang, G. Lu, C. Qiao, Y. Hu, K.-Y. Yuen, Q. Wang, H. Zhou, J. Yan, J. Qi, Structural and functional basis of SARS-CoV-2 entry by using human ACE2. *Cell* **181**, 894–904.e9 (2020). doi:10.1016/j.cell.2020.03.045 Medline

18. J. Lan, J. Ge, J. Yu, S. Shan, H. Zhou, S. Fan, Q. Zhang, X. Shi, Q. Wang, L. Zhang, X. Wang, Structure of the SARS-CoV-2 spike receptor-binding domain bound to the ACE2 receptor. *Nature* **581**, 215–220 (2020). doi:10.1038/s41586-020-2180-5 Medline

19. C. Toelzer, K. Gupta, S. K. N. Yadav, U. Borucu, A. D. Davidson, M. Kavanagh Williamson, D. K. Shoemark, F. Garzoni, O. Staufer, R. Milligan, J. Capin, A.

J. Mulholland, J. Spatz, D. Fitzgerald, I. Berger, C. Schaffitzel, Free fatty acid binding pocket in the locked structure of SARS-CoV-2 spike protein. *Science* **370**, 725–730 (2020). doi:10.1126/science.abd3255 Medline

20. C. Gu, X. Cao, Z. Wang, X. Hu, Y. Yao, Y. Zhou, P. Liu, X. Liu, G. Gao, X. Hu, Y. Zhang, Z. Chen, L. Gao, Y. Peng, F. Jia, C. Shan, L. Yu, K. Liu, N. Li, W. Guo, G. Jiang, J. Min, J. Zhang, L. Yang, M. Shi, T. Hou, Y. Li, W. Liang, G. Lu, C. Yang, Y. Wang, K. Xia, Z. Xiao, J. Xue, X. Huang, X. Chen, H. Ma, D. Song, Z. Pan, X. Wang, H. Guo, H. Liang, Z. Yuan, W. Guan, S.-J. Deng, A human antibody of potent efficacy against SARS-CoV-2 in rhesus macaques showed strong blocking activity to B.1.351. *MAbs* **13**, 1930636 (2021). doi:10.1080/19420862.2021.1930636 Medline

21. Y. Yan, S. Mukherjee, K. G. Harikumar, T. S. Strutzenberg, X. E. Zhou, K. Suino-Powell, T.-H. Xu, R. D. Sheldon, J. Lamp, J. S. Brunzelle, K. Radziwon, A. Ellis, S. J. Novick, I. E. Vega, R. G. Jones, L. J. Miller, H. E. Xu, P. R. Griffin, A. A. Kossiakoff, K. Melcher, Structure of an AMPK complex in an inactive, ATP-bound state. *Science* **373**, 413–419 (2021). doi:10.1126/science.abe7565 Medline

22. S. Kumar, A. Chandele, A. Sharma, Current status of therapeutic monoclonal antibodies against SARS-CoV-2. *PLOS Pathog.* **17**, e1009885 (2021). doi:10.1371/journal.ppat.1009885 Medline

23. S. Q. Zheng, E. Palovcak, J.-P. Armache, K. A. Verba, Y. Cheng, D. A. Agard, MotionCor2: Anisotropic correction of beam-induced motion for improved cryo-electron microscopy. *Nat. Methods* **14**, 331–332 (2017). doi:10.1038/nmeth.4193 Medline

24. A. Rohou, N. Grigorieff, CTFFIND4: Fast and accurate defocus estimation from electron micrographs. *J. Struct. Biol.* **192**, 216–221 (2015). doi:10.1016/j.jsb.2015.08.008 Medline

25. J. Zivanov, T. Nakane, B. O. Forsberg, D. Kimanius, W. J. H. Hagen, E. Lindahl, S. H. W. Scheres, New tools for automated high-resolution cryo-EM structure determination in RELION-3. *eLife* **7**, e42166 (2018). doi:10.7554/eLife.42166 Medline

26. A. Punjani, J. L. Rubinstein, D. J. Fleet, M. A. Brubaker, cryoSPARC: Algorithms for rapid unsupervised cryo-EM structure determination. *Nat. Methods* **14**, 290–296 (2017). doi:10.1038/nmeth.4169 Medline

27. R. Sanchez-Garcia, J. Gomez-Blanco, A. Cuervo, J. M. Carazo, C. O. S. Sorzano, J. Vargas, DeepEMhancer: A deep learning solution for cryo-EM volume post-processing. *Commun. Biol.* **4**, 874 (2021). doi:10.1038/s42003-021-02399-1 Medline

28. T. Schwede, J. Kopp, N. Guex, M. C. Peitsch, SWISS-MODEL: An automated protein homology-modeling server. *Nucleic Acids Res.* **31**, 3381–3385 (2003). doi:10.1093/nar/gkg520 Medline

29. T. Zhou, Y. Tsybovsky, J. Gorman, M. Rapp, G. Cerutti, G.-Y. Chuang, P. S. Katsamba, J. M. Sampson, A. Schön, J. Bimela, J. C. Boyington, A. Nazzari, A. S. Olia, W. Shi, M. Sastry, T. Stephens, J. Stuckey, I.-T. Teng, P. Wang, S. Wang, B. Zhang, R. A. Friesner, D. D. Ho, J. R. Mascola, L. Shapiro, P. D. Kwong, Cryo-EM structures of SARS-CoV-2 Spike without and with ACE2 reveal a pH-dependent switch to mediate endosomal positioning of receptor-binding domains. *Cell Host Microbe* **28**, 867–879.e5 (2020). doi:10.1016/j.chom.2020.11.004 Medline

30. A. C. Walls, Y.-J. Park, M. A. Tortorici, A. Wall, A. T. McGuire, D. Veesler, Structure, function, and antigenicity of the SARS-CoV-2 Spike glycoprotein. *Cell* **181**, 281–292.e6 (2020). doi:10.1016/j.cell.2020.02.058 Medline

31. E. F. Pettersen, T. D. Goddard, C. C. Huang, G. S. Couch, D. M. Greenblatt, E. C. Meng, T. E. Ferrin, UCSF Chimera--A visualization system for exploratory research and analysis. *J. Comput. Chem.* **25**, 1605–1612 (2004). doi:10.1002/jcc.20084 Medline

32. P. Emsley, K. Cowtan, Coot: Model-building tools for molecular graphics. *Acta Crystallogr. D Biol. Crystallogr.* **60**, 2126–2132 (2004). doi:10.1107/S0907444904019158 Medline

33. P. D. Adams, K. Gopal, R. W. Grosse-Kunstleve, L.-W. Hung, T. R. Ioerger, A. J. McCoy, N. W. Moriarty, R. K. Pai, R. J. Read, T. D. Romo, J. C. Sacchettini, N. K. Sauter, L. C. Storoni, T. C. Terwilliger, Recent developments in the PHENIX software for automated crystallographic structure determination. *J. Synchrotron Radiat.* **11**, 53–55 (2004). doi:10.1107/S0909049503024130 Medline

34. T. I. Croll, ISOLDE: A physically realistic environment for model building into low-resolution electron-density maps. *Acta Crystallogr. D Struct. Biol.* **74**, 519–530 (2018). doi:10.1107/S2059798318002425 Medline

35. E. F. Pettersen, T. D. Goddard, C. C. Huang, E. C. Meng, G. S. Couch, T. I. Croll, J. H. Morris, T. E. Ferrin, UCSF ChimeraX: Structure visualization for researchers, educators, and developers. *Protein Sci.* **30**, 70–82 (2021). doi:10.1002/pro.3943 Medline

36. K. Melcher, L.-M. Ng, X. E. Zhou, F.-F. Soon, Y. Xu, K. M. Suino-Powell, S.-Y. Park, J. J. Weiner, H. Fujii, V. Chinnusamy, A. Kovach, J. Li, Y. Wang, J. Li, F. C. Peterson, D. R. Jensen, E.-L. Yong, B. F. Volkman, S. R. Cutler, J.-K. Zhu, H. E. Xu, A gate-latch-lock mechanism for hormone signalling by abscisic acid receptors. *Nature* **462**, 602–608 (2009). doi:10.1038/nature08613 Medline
